# Identification of copper (Cu) stress-responsive grapevine microRNAs and their target genes by high-throughput sequencing

**DOI:** 10.1098/rsos.180735

**Published:** 2019-01-23

**Authors:** Songtao Jiu, Xiangpeng Leng, Muhammad Salman Haider, Tianyu Dong, Le Guan, Zhenqiang Xie, Xiaopeng Li, Lingfei Shangguan, Jinggui Fang

**Affiliations:** 1 Key Laboratory of Genetics and Fruit development, College of Horticulture, Nanjing Agricultural University, Nanjing, Jiangsu Province, People's Republic of China; 2 Department of Plant Science, School of Agriculture and Biology, Shanghai Jiao Tong University, Shanghai, People's Republic of China; 3 College of Horticulture, Qingdao Agricultural University, Qingdao, Shandong Province, People's Republic of China

**Keywords:** grapevine, microRNAs, Cu stress, high-throughput sequencing

## Abstract

MicroRNAs (miRNAs) are a class of single-stranded non-coding small RNAs (sRNAs) that are 20–24 nucleotides (nt) in length. Extensive studies have indicated that miRNAs play important roles in plant growth, development and stress responses. With more copper (Cu) and copper containing compounds used as bactericides and fungicides in plants, Cu stress has become one of the serious environmental problems that affect plant growth and development. In order to uncover the hidden response mechanisms of Cu stress, two small RNA libraries were constructed from Cu-treated and water-treated (Control) leaves of ‘Summer Black’ grapevine. Following high-throughput sequencing and filtering, a total of 158 known and 98 putative novel miRNAs were identified in the two libraries. Among these, 100 known and 47 novel miRNAs were identified as differentially expressed under Cu stress. Subsequently, the expression patterns of nine Cu-responsive miRNAs were validated by quantitative real-time PCR (qRT-PCR). There existed some consistency in expression levels of Cu-responsive miRNAs between high throughput sequencing and qRT-PCR assays. The targets prediction of miRNAs indicates that miRNA may regulate some transcription factors, including AP2, SBP, NAC, MYB and ARF during Cu stress. The target genes for two known and two novel miRNAs showed specific cleavage sites at the 10th and/or 11th nucleotide from the 5′-end of the miRNA corresponding to their miRNA complementary sequences. The findings will lay the foundation for exploring the role of the regulation of miRNAs in response to Cu stress and provide valuable gene information for breeding some Cu-tolerant grapevine cultivars.

## 1. Introduction

MicroRNAs (miRNAs) are endogenous gene regulators distributed widely in plant genomes, and they play critical roles in plant growth, development, signal transduction and stress responses [1–5]. According to miRBase (http://www.mirbase.org/, V22, March 2018), thousands of miRNAs have been identified and characterized in plants, animals and viruses [6]. The identification of miRNAs is of great significance in the acquisition of insights into sRNA-based regulatory functions along with numerous conserved miRNAs being identified by conventional (Sanger) sequencing approaches [7]. Nevertheless, most species-specific or tissue-specific miRNAs are difficult to perceive, probably due to their low accumulation and/or the insufficient stringency of the sequencing method [8–10]. The advent of new sequencing technologies has made it possible to mine even species-/tissue-specific miRNAs with low abundance, which have been successfully used in many crops such as, *Arabidopsis*, rice, poplar, clover, cotton, maize*,* peanut, tomato, citrus and grapevine [11–22].

Cu is an essential micronutrient for plant growth and plays a key role in many physiological processes including photosynthesis, hormone perception, cell wall metabolism, respiratory electron transport and oxidative stress responses. Nevertheless, it can be highly toxic at supra-optimal amounts and cause the rapid accumulation of reactive oxygen species (ROS), which affect a wide range of biochemical and physiological processes by damaging nucleic acids, oxidizing proteins, and causing lipid peroxidation, in animals and humans as well as plants [23,24]. It has been previously reported that plants develop an important regulatory mechanism to adapt to the frequently changing availability of Cu [25]. In China, due to long-term waste-water irrigation, over-use of Cu-containing fungicides and pesticides in plants, Cu stress has become one of the detrimental environmental crises that seriously affects plant growth and development. Thus, researchers have turned their attention to studying the mechanism of Cu tolerance in plants. To date, studies on miRNAs transcription in response to Cu stress have mainly focused on model plants such as *A. thaliana* and *Populus tremula* [25,26]. However, limited works have been reported on grapevine miRNAs in response to Cu stress.

Grapevine (*V. vinifera* L.) is one of the most economically important fruit crops worldwide and has nutritional and processing properties, but is extremely vulnerable to Cu stress [27]. In recent years, small RNA (sRNA) sequencing has demonstrated the identification of a wide range of vvi-miRNAs from different grapevine cultivars and their hybrids [8,19–22]. To date, 186 grapevine miRNAs have been deposited into the miRBase [6]. However, a systematic report on the characterization of Cu-responsive miRNAs and their proposed target genes in grapes is still lacking. In this study, we report the identification of known and putative novel miRNAs involved in response to Cu stress. Firstly, to gain precise knowledge of Cu stress-responsive miRNAs and their target genes, two sRNA libraries were generated from Cu-treated and water-treated (control) leaves of the ‘Summer Black’ grape cultivar (*V. vinifera* × *V. labrusca*). Genome-wide identification of Cu-responsive miRNAs was performed based on published grape genome sequences and deep sequencing technology [28]. The expression pattern of Cu-responsive miRNAs and its putative targets were analysed by qRT-PCR assay, which will be helpful to understanding the molecular mechanisms of grapevine miRNAs in response to Cu stress. Furthermore, this study will provide a foundation for exploring the role of the regulation of miRNAs in response to Cu stress and also provide valuable gene information for breeding Cu-tolerant grapevine cultivars.

## 2. Material and methods

### 2.1. Plant materials and Cu treatment

Two-year-old ‘Summer Black’ grapevine (hybrids of *V. vinifera* and *V. labrusca*) trees grown under the standard field conditions at the Nanjing Agricultural University fruit farm, Nanjing, China were chosen as the experimental material; 100 µM concentration of CuSO_4_ was chosen as the Cu stress treatment because it can lead to a drastic decrease of several physiological and growth traits in preliminary experiments. Twenty-four hours after treatment, mixed ‘Summer Black’ young grapevine young leaves (two weeks old), large leaves (five weeks old), and old leaves (nine weeks old) in randomly-selected plants from both the Cu-treated and control groups were collected for high-throughput sequencing. Each style sample has three biological replications. Additionally, the third leaf (five weeks old) from the stem apex was harvested at 0, 6 12 and 24 h after treatment for qRT-PCR assay. To reduce the differences between individuals, leaf samples were collected from six grapevine seedlings at a single time point, and there were three biological replications per treatment. All the samples were immediately frozen in liquid nitrogen and stored at −80°C until use.

### 2.2. Small RNA library construction and deep sequencing

The mixed leaf samples from ‘Summer Black’ grapevine at three different growth stages, young leaves (two weeks old), large leaves (five weeks old), and old leaves (nine weeks old) treated with CuSO_4_, were used for RNA extraction. The total RNA samples were first extracted using our modified CTAB method [21]. Isolation of small RNAs and preparation of small RNA libraries were performed based on the procedure of Wang *et al.* [21]. sRNAs were first separated from the total RNA by size fractionation with 15% TBE urea polyacrylamide gel (TBU) and small RNA regions corresponding to the 18–30 nucleotide bands in the marker lane were excised and recovered. The 18–30 nt small RNAs were 5′ and 3′ RNA adapter-ligated by T4 RNA ligase and at each step, length validated and purified by TBU electrophoretic separation. The adapter-ligated small RNA was subsequently transcribed into cDNA by SuperScript II Reverse Transcriptase (Invitrogen) and PCR amplified using primers that annealed to the ends of the adapters. The amplified cDNA constructs were purified and recovered. The purified cDNA from the two sRNA libraries were sequenced with an Illumina Genome Analyzer II (LC Sciences, Hangzhou, China) according to the manufacturer's protocols.

### 2.3. Identification of known and novel miRNAs in grapevine

To identify known and novel miRNAs in grapevine, the raw sequences were processed as described by Sunkar *et al.* [9]. All sRNAs sequences from 18 nt to 30 nt obtained were removed from the vector sequences, then the modified sequences were further subjected to removal of rRNA, tRNA, snRNA, snoRNA and those containing the polyA tails, and finally the remaining sequences were compared against known plant miRNAs in the miRBase [29]. A maximum of one mismatch in the first 16 nt of the miRNA and three mismatches in total between the target miRNAs and known miRNAs deposited in the miRBase database were considered as known vvi-miRNAs. To study novel miRNA precursor sequences, all sRNAs from grapevine were aligned against the grapevine genome and then the miRNA candidates were processed by miRCat (http://srna-tools.cmp.uea.ac.uk/) [29], using default parameters, to generate the secondary structures (electronic supplementary material, figures S1 and S2).

### 2.4. Differential expression analysis of miRNAs under copper stress

To investigate the differentially expressed miRNAs between libraries, we compared the gene expression patterns of miRNAs in CK and Cu treatment libraries. To this end, we considered the following criteria: (i) adjusted *p*-value should be less than 0.01 (*p*-value < 0.01) in at least one dataset; (ii) fold change or log_2_ ratio of normalized counts between Cu and CK libraries was greater than 1 or less than −1 in one of the libraries.

Afterwards, the fold-change between Cu treatment and CK and *p*-value were calculated from the normalized expression using the formula shown below: Fold-change formula: Fold change = log_2_ (Cu/CK)

*P*-value formula:

$$\left(\frac{x}{y}\right)={\left(\frac{N_{2}}{N_{1}}\right)}^{Y}{\frac{(x+y)!}{x!y!{\left(1+(N_{2}/N_{1})\right)}^{\left(x+y+1\right)}}}_{D(y\geq y_{\max}|x)\;=\sum_{y\geq y_{\max}}^{\infty }\;p(y/x)}^{C(y\leq y_{\min}|x)\;=\sum_{y=0}^{y\leq y_{\min}}\;p(y/x)}$$

where *N*_1_ and *N*_2_ are sampling size (total reads of two libraries respectively), *x* and *y* are reads of specific miRNA in two libraries respectively. To compute the confidence intervals, we made use of the cumulative distributions:

$$C(y\leq y_{\min}|x)=\overset{y\leq y_{\min}}{\underset{y=0}{\sum }}p\left(\frac{y}{x}\right)\;\mathrm{and}\;\;D(y\geq y_{\max}|x)=\overset{\infty }{\underset{y\geq y_{\max}}{\sum }}p\left(\frac{y}{x}\right)$$

which allow the computation of an interval [*y*_min_, *y*_max_]*_ε_* and serve as a significance test when comparing [30].

A Poisson distribution model was used for estimating the statistical significance of miRNA expression changes under control and treatment conditions [30]. Upregulation of any miRNA expression levels was considered a positive value while negative values indicated downregulation. Clustering analysis was performed using Cluster 3.0, and the heat map was visualized using Heatmap builder and TreeView [31].

### 2.5. Prediction of potential target mRNAs for miRNAs in grapevine

Target predictions were performed based on methods described by Allen *et al.* and Schwab *et al.* [11,32]. Putative miRNAs were first blasted against the grapevine unigene database on the Genoscope (http://www.genoscope.cns.fr/). BLASTn hits possessing less than four mismatches were chosen as the candidate targets, and then BLASTx was used to obtain their putative functions. The predicted targets and their functions are shown in electronic supplementary material, tables S1 and S2.

### 2.6. qRT-PCR verification

Small RNA was extracted from ‘Summer Black’ grapevine leaves sprayed with 100 µM CuSO_4_ treatment for 0, 6, 12 and 24 h using the mirVana miRNA Isolation Kit (Ambion) according to the manufacturer's protocol. According to the poly (A) polymerase procedure (Ambion), small RNA was ligated to poly (A) tails. Using T4 RNA ligase (Invitrogen, Carlsbad, CA), 5′ adaptor (5′-CGACUGGAGCACGAGGACACUGACAUGGACUGAAGGAGUAGAAA-3′) was added to the poly (A)-tailed RNA. Small RNA and RT primer (ATTCTAGAGGCCGAGGCGGCCGACATG-d [T]_30_ [A, G or C] [A, G, C or T]) were used to amplify the cDNA by reverse transcription [21]. The products were used as templates to analyse the expression of the miRNAs. Meanwhile, total RNA was extracted and used to detect the expression amounts of the target genes. qRT-PCR was conducted with the Rotor-Gene 3000 (Corbett Robotics, Australia) and the Rotor-Gene software version 6.1. For each reaction, 1 µl of diluted cDNA (equivalent to about 100 pg of total RNA) was mixed with 10 µl of 2X SYBR green reaction mix (SYBR^®^ Green qRT-PCR Master Mix; Toyobo, Osaka, Japan), and 5 pmol each of the forward and the reverse primers were added in a final volume of 20 µl. The conditions for the PCR amplification were as follows: initial denaturation at 95°C for 2 min, followed by 50 cycles of denaturation at 95°C for 30 s, annealing at 60°C for 20 s, and extension at 72°C for 20 s. All reactions were repeated three times, and the homologous genes of the *Arabidopsis 5.8S rRNA* in grapevine and *Actin* were respectively used as reference genes in the qRT-PCR detection of miRNAs and target genes. The primers were listed in electronic supplementary material, tables S3 and S4. The relative expression amounts of the miRNAs and target genes were calculated using the 2^−△△Ct^ method [33].

### 2.7. Mapping of mRNA cleavage sites using 5′-RLM-RACE

Total RNA was respectively extracted from 200 mg of young leaves from ‘Summer Black’ grapevine using TRIzol reagent (Invitrogen, Life Technologies, Carlsbad, CA). Genomic DNA was removed by 15 min incubation at 37°C with RNase-Free DNase (TaKaRa, Otsu, Japan) followed by an RNA Clean Purification Kit (BioTeke, Beijing, China). The low molecular weight (LMW) RNA and high molecular weight (HMW) RNAs were separated with 10 M LiCl [34]. After construction of libraries of poly(A)-tailed HMW RNA and adapter-ligated HMW RNA, the productions of reverse transcription of poly(A)-tailed HMW RNA and adapter-ligated HMW RNA were performed with RLM-RACE using a corresponding common primer and specific primers (electronic supplementary material, table S5). The amplification products were gel purified, cloned, and sequenced, and at least eight independent clones were sequenced.

### 2.8. GO, KEGG and network analysis

GO analysis was used to study the functions of the target genes of the miRNAs based on the database (http://www.geneontology.org/). KEGG pathway analysis of the targets was performed simultaneously based on the KEGG database (http://www.genome.jp/kegg/). The results were filtered based on a Fisher Exact statistic methodology similar to that previously described [35,36]. The network between miRNAs and their target genes was subsequently assembled according to the GO analysis results.

## 3. Results

### 3.1. Characterization of the miRNAs from deep sequencing of grapevine sRNA libraries

The high throughput sequencing has generated a total of 9 856 414 and 10 581 133 raw reads from the sRNA libraries constructed from Cu treatment and the control, respectively. After the removal of ambiguous, adaptor, insert, polyA, and RNAs < 18 nt in length from raw reads, a sum of 5 488 559 (Cu treatment) and 6 031 513 (control) clean reads were mapped to the grapevine genome published in 2007 [28], and miRNA, tRNA, siRNA, snRNA, snoRNA, rRNA, repeat regions, exon and intron RNA reads were annotated. In addition, 2 833 183 and 3 843 252 un-annotated reads were used for the prediction of novel miRNAs in treatment and the control, respectively. The number and percentage of various sRNAs were listed in table 1.

**Table 1.** Distribution of small RNAs among different categories in control and Cu treatment.

	control				Cu treatment			
category	unique	per cent (%)	total	per cent (%)	unique	per cent (%)	total	per cent (%)
Exon_antisense	22 940	1.59	79 175	0.75	14 435	1.18	55 404	0.56
Exon_sense	109 005	7.57	248 648	2.35	167 547	13.72	262 235	2.66
Intron_antisense	15 807	1.10	42 934	0.41	12 376	1.01	27 846	0.28
Intron_sense	38 909	2.70	315 737	2.98	39 783	3.26	194 142	1.97
Known_miRNA	387	0.03	593 973	5.61	199	0.02	89 944	0.91
miRNA	691	0.05	161 289	1.52	537	0.04	45 848	0.47
rRNA	163 779	11.37	4 315 618	40.79	179 248	14.68	5 461 082	55.41
rRNAetc	647	0.04	144 070	1.36	652	0.05	49 670	0.50
repeat	171 017	11.87	473 544	4.48	121 279	9.93	289 022	2.93
snRNA	3755	0.26	22 947	0.22	4445	0.36	19 357	0.20
snoRNA	3442	0.24	16 362	0.15	5116	0.42	40 209	0.41
tRNA	19 338	1.34	323 584	3.06	21 336	1.75	488 472	4.96
unann	890 981	61.84	3 843 252	36.32	653 826	53.56	2 833 183	28.74
mapping to genome	639 803	44.41	6 031 513	57	643 170	52.69	5 488 559	55.69
total	1 440 698	100	10 581 133	100	1 220 779	100	9 856 414	100

The length distribution of the sRNAs from the two different libraries ranged from 10 to 40 nt, as shown in electronic supplementary material, figure S3. Moreover, the sRNAs with length of 21 nt and 24 nt were the two main sRNA classes among the sequences of control library, and the 21 nt sRNA was the most abundant category, in agreement with previous reports in grapevine and tomato [12,17,22,28], but different from those reported in *Arabidopsis*, rice and peanut [37–40]. These cases suggested that some differences might exist in the sRNA biogenesis pathways in various plants. In addition, analysis of the first nucleotide of 19–24 nt long sRNAs indicated that many sRNAs started with a uridine (U) at their 5′-ends and most of them are 21 nt, 22 nt and 23 nt in length, with the former being most outstanding in number (electronic supplementary material, figure S4). However, the proportion of 21–32 nt sequences was high in the Cu stress library, and 24 nt sRNA was the most abundant category, occupying 13.70% of the total (electronic supplementary material, figure S3). Most importantly, the 25–32 nt sRNA sequences are induced obviously by Cu stress.

### 3.2. Identification of known miRNAs in grapevine

The clean reads generated from two grapevine sRNA libraries were aligned against mature plant miRNAs and the precursors in miRBase 22.0 (March 2018), allowing for a maximum of one mismatch in the first 16 nt of the miRNA and three mismatches in total between the target miRNAs and the known miRNAs. Finally, 158 known miRNAs were identified in the two libraries, belonging to 46 miRNA families. The vast majority of the recognized miRNA families, for example, miR156, miR159, miR167, miR394 and miR398, are highly conserved in a variety of plant species. Furthermore, as expected, we also found several known but non-conserved miRNAs, such as miR2111, miR477 and miR2950, in our datasets. They were previously identified only from one or a few plant species. Interestingly, the number of miRNAs varied remarkably among different families (electronic supplementary material, figure S5). The largest family was the miR169 family with 23 members, including two members only identified in the control library and the remaining 21 presented in both libraries, followed by the miR395 family with 13 members. However, the miR159, miR162, miR168, miR172, miR2111, miR2950, miR390, miR397, miR408, miR479, miR482 and miR828 families were the smallest, each containing only one miRNA member. In addition, the miR397 family was exclusively detected in the control library. One member of the miR3624 family and the miR3638 family were exclusively detected in the Cu stress library. In addition, 145 known miRNAs from Cu and control libraries, two known miRNAs (vvi-miR3624-5p and vvi-miR3638-3p) were only found in the Cu library; 11 known miRNAs that were vvi-miR156a, vvi-miR169d, vvi-miR169u, vvi-miR3625-5p, vvi-miR3631b-3p, vvi-miR3635-5p, vvi-miR397a, vvi-miR399b, vvi-miR399c, vvi-miR399i, vvi-miR477b-5p and vvi-miR3624-5p were specific to the control library (electronic supplementary material, figure S6a). Among the known miRNAs, the vvi-miR166 family had the most abundant reads accounting for 94.2% of all the known miRNA reads. In this family, the number of vvi-miR166h reads was over 100 000 in the two libraries, followed by the vvi-miR3634, vvi-miR162, vvi-miR159, vvi-miR482 and vvi-miR403 families, whose redundancies were more than ten thousand. However, other miRNA families such as vvi-miR169, vvi-miR172, vvi-miR2111, vvi-miR395, vvi-miR3630, vvi-miR3637 vvi-miR398 and vvi-miR399 had a small number (less than 10) reads. Interestingly, the number and the abundance level of different miRNA families between the control and Cu treatment libraries exhibited significant divergence (table 2), which in turn could reflect the discrepancy in their potential functions during the grapevine's response to Cu.

**Table 2.** The differentially-expressed of known miRNAs identified from grapevine in response to Cu stress. -std, standardized expression of miRNAs in sample; **, | log_2_FC| ≥ 1 and *p*-value < 0.01, *, | log_2_FC| ≥ 1 and 0.01 < *p*-value < 0.05, hollow, no obvious difference; S, the sequences were the same between sequencing and miRBase database. *D*, the sequences were different between sequencing and miRBase database. ↑,↓denote upregulated, downregulated.

miR-name	mature sequences	control	CK-std	Cu-treated	Cu-std	fold-change (log_2_ Cu/CK)	*p*-value	sig-lable	S/D	regulated
vvi-miR156b	TGACAGAAGAGAGTGAGCAC	60	5.6705	14	1.4204	−1.9971787	1.96 × 10^−7^	**	S	↓
vvi-miR156c	TGACAGAAGAGAGTGAGCAC	60	5.6705	14	1.4204	−1.9971787	1.96 × 10^−7^	**	S	↓
vvi-miR156d	TGACAGAAGAGAGTGAGCAC	62	5.8595	14	1.4204	−2.0444803	7.81 × 10^−8^	**	S	↓
vvi-miR156f	TTGACAGAAGATAGAGAGCAC	357	33.7393	110	11.1602	−1.5960672	5.67 × 10^−28^	**	S	↓
vvi-miR156g	TTGACAGAAGATAGAGAGCAC	357	33.7393	110	11.1602	−1.5960672	5.67 × 10^−28^	**	S	↓
vvi-miR156i	TTGACAGAAGATAGAGAGCAC	357	33.7393	110	11.1602	−1.5960672	5.67 × 10^−28^	**	S	↓
vvi-miR159c	TTTGGATTGAAGGGAGCTCTA	35 427	3348.1292	5406	548.4753	−2.6098566	0	**	S	↓
vvi-miR160a	TGCCTGGCTCCCTGAATGCCA	31	2.9297	4	0.4058	−2.8519122	5.17 × 10^−6^	**	D	↓
vvi-miR160b	TGCCTGGCTCCCTGAATGCCA	31	2.9297	4	0.4058	−2.8519122	5.17 × 10^−6^	**	D	↓
vvi-miR160c	TGCCTGGCTCCCTGTATGCCA	608	57.4608	120	12.1748	−2.23868	3.04 × 10^−72^	**	S	↓
vvi-miR160d	TGCCTGGCTCCCTGTATGCCA	612	57.8388	120	12.1748	−2.2481396	4.46 × 10^−73^	**	S	↓
vvi-miR160e	TGCCTGGCTCCCTGTATGCCA	612	57.8388	120	12.1748	−2.2481396	4.46 × 10^−73^	**	S	↓
vvi-miR162	TCGATAAACCTCTGCATCCAG	6683	631.5959	2387	242.1773	−1.3829382	0	**	S	↓
vvi-miR164a	TGGAGAAGCAGGGCACGTGCA	495	46.7814	144	14.6098	−1.6789986	6.61 × 10^−41^	**	S	↓
vvi-miR164c	TGGAGAAGCAGGGCACGTGCA	499	47.1594	146	14.8127	−1.6707107	6.01 × 10^−41^	**	S	↓
vvi-miR164d	TGGAGAAGCAGGGCACGTGCA	495	46.7814	144	14.6098	−1.6789986	6.61 × 10^−41^	**	S	↓
vvi-miR166a	TCTCGGACCAGGCTTCATTCC	111 537	10 541.121	19 072	1934.9837	−2.4456349	0	**	D	↓
vvi-miR166b	TCGGACCAGGCTTCATTCCTC	34 146	3227.0646	5723	580.6371	−2.4745138	0	**	D	↓
vvi-miR166c	TCGGACCAGGCTTCATTCCCC	542 085	51 231.281	81 080	8226.1155	−2.6387417	0	**	S	↓
vvi-miR166d	TCGGACCAGGCTTCATTCCCC	543 446	51 359.906	81 262	8244.5806	−2.6391246	0	**	S	↓
vvi-miR166e	TCGGACCAGGCTTCATTCCCC	542 085	51 231.281	81 080	8226.1155	−2.6387417	0	**	S	↓
vvi-miR166f	TCGGACCAGGCTTCATTCCCC	546 903	51 686.62	82 300	8349.8928	−2.6299613	0	**	S	↓
vvi-miR166g	TCGGACCAGGCTTCATTCCCC	548 394	51 827.531	82 682	8388.6493	−2.6272082	0	**	S	↓
vvi-miR166h	TCGGACCAGGCTTCATTCCCC	652 744	61 689.424	100 147	10 160.592	−2.6020387	0	**	S	↓
vvi-miR167a	TGAAGCTGCCAGCATGATCTGG	1153	108.9675	218	22.1176	−2.3006312	6.05 × 10^−140^	**	D	↓
vvi-miR167b	TGAAGCTGCCAGCATGATCTA	1142	107.928	284	28.8137	−1.9052424	2.55 × 10^−109^	**	S	↓
vvi-miR167c	TGAAGCTGCCAGCATGATCTC	239	22.5874	58	5.8845	−1.9405265	9.95 × 10^−25^	**	S	↓
vvi-miR167d	TGAAGCTGCCAGCATGATCTA	576	54.4365	133	13.4938	−2.0122776	4.42 × 10^−60^	**	S	↓
vvi-miR167e	TGAAGCTGCCAGCATGATCTA	1057	99.8948	214	21.7118	−2.2019302	8.34 × 10^−122^	**	S	↓
vvi-miR168	TCGCTTGGTGCAGGTCGGGAA	1123	106.1323	450	45.6555	−1.2170032	1.27 × 10^−56^	**	S	↓
vvi-miR169e	TAGCCAAGGATGACTTGCCTG	16	1.5121	1	0.1015	−3.8970019	0.0002538	**	D	↓
vvi-miR169f	CAGCCAAGGATGACTTGCCGA	44	4.1583	8	0.8117	−2.3569753	8.64 × 10^−7^	**	S	↓
vvi-miR169 g	CAGCCAAGGATGACTTGCCGA	47	4.4419	9	0.9131	−2.2823321	5.94 × 10^−7^	**	S	↓
vvi-miR169l	TGAGCCAAGGATGACTTGCCG	16	1.5121	2	0.2029	−2.8977128	0.0012357	**	D	↓
vvi-miR169n	AATAGAGCCAAGGATGACTTGC	12	1.1341	5	0.5073	−1.1606368	0.1303276		D	
vvi-miR169q	AATAGAGCCAAGGATGACTTGC	12	1.1341	5	0.5073	−1.1606368	0.1303276		D	
vvi-miR169r	TGAGTCAAGGATGACTTGCCGA	19	1.7956	1	0.1015	−4.1449144	4.08 × 10^−5^	**	D	↓
vvi-miR169t	CGAGTCAAGGATGACTTGCCGA	11	1.0396	3	0.3044	−1.7719883	0.0487776	*	D	↓
vvi-miR169v	AAGCCAAGGATGAATTGCCGG	78	7.3716	21	2.1306	−1.790718	3.27 × 10^−8^	**	S	↓
vvi-miR171a	TGATTGAGCCGTGCCAATATC	117	11.0574	34	3.4495	−1.680553	8.51 × 10^−11^	**	S	↓
vvi-miR171b	TTGAGCCGCGTCAATATCTCC	47	4.4419	7	0.7102	−2.6448797	5.48 × 10^−8^	**	D	↓
vvi-miR171c	TGATTGAGCCGTGCCAATATC	158	14.9322	52	5.2758	−1.500965	3.39 × 10^−12^	**	S	↓
vvi-miR171d	TGATTGAGCCGTGCCAATATC	158	14.9322	52	5.2758	−1.500965	3.39 × 10^−12^	**	S	↓
vvi-miR171i	TGATTGAGCCGTGCCAATATC	117	11.0574	34	3.4495	−1.680553	8.51 × 10^−11^	**	S	↓
vvi-miR171j	TGATTGAGCCGTGCCAATATC	158	14.9322	52	5.2758	−1.500965	3.39 × 10^−12^	**	S	↓
vvi-miR172d	AGAATCTTGATGATGCTGCAT	28	2.6462	9	0.9131	−1.5350773	0.0033832	**	D	↓
vvi-miR2111-5p	TAATCTGCATCCTGAGGTCTA	13	1.2286	3	0.3044	−2.012975	0.0188862	*	S	↓
vvi-miR2950-5p	TTCCATCTCTTGCACACTGGA	65	6.143	15	1.5219	−2.0130698	5.11 × 10^−8^	**	S	↓
vvi-miR319b	TTGGACTGAAGGGAGCTCCC	608	57.4608	266	26.9875	−1.0902867	1.23 × 10^−26^	**	D	↓
vvi-miR319c	TTGGACTGAAGGGAGCTCCC	607	57.3663	266	26.9875	−1.0879121	1.66 × 10^−26^	**	D	↓
vvi-miR319f	TTGGACTGAAGGGAGCTCCC	607	57.3663	266	26.9875	−1.0879121	1.66 × 10^−26^	**	D	↓
vvi-miR319 g	TTGGACTGAAGGGAGCTCCC	426	40.2603	147	14.9141	−1.432681	1.88 × 10^−28^	**	D	↓
vvi-miR3623-3p	TGGTGCTTGGACGAATTTGCT	1154	109.062	557	56.5114	−0.9485347	2.22 × 10^−39^		D	
vvi-miR3623-5p	TCACAAGTTCATCCAAGCACCA	3431	324.2564	1694	171.8678	−0.9158358	3.51 × 10^−107^		S	
vvi-miR3624-3p	TCAGGGCAGCAGCATACTACT	21	1.9847	80	8.1165	2.0319368	1.87 × 10^−10^	**	S	↑
vvi-miR3625-3p	CGGGAGATGACTACTGGAAGC	15	1.4176	3	0.3044	−2.2194103	0.0070284	**	S	↓
vvi-miR3626-3p	TCAATTTCACAGCGACCACTG	34	3.2133	11	1.116	−1.5257187	0.0012548	**	D	↓
vvi-miR3626-5p	CCGGTAGTCGCTGTGAAATTG	43	4.0638	19	1.9277	−1.0759489	0.005448	**	D	↓
vvi-miR3627-5p	TTGTCGCAGGAGAGACGGCACT	13	1.2286	16	1.6233	0.4019144	0.4578508		S	
vvi-miR3629a-3p	TGGCTGCTGAGAAAATGTAGG	44	4.1583	8	0.8117	−2.3569753	8.64 × 10^−7^	**	D	↓
vvi-miR3629b	TGGCTGCTGAGAAAATGTAGG	42	3.9693	8	0.8117	−2.2898661	2.34 × 10^−6^	**	D	↓
vvi-miR3629c	TGGCTGCTGAGAAAATGTAGG	44	4.1583	8	0.8117	−2.3569753	8.64 × 10^−7^	**	D	↓
vvi-miR3630-3p	GAGAATGATGATTTGTCTTTGGGAATCTCTCTGATG	78	7.3716	209	21.2045	1.5243208	2.83 × 10^−17^	**	D	↑
vvi-miR3630-5p	GCAAGTGACGATATCAGACA	3	0.2835	10	1.0146	1.8394904	0.0420643	*	D	↑
vvi-miR3632-3p	TTTCCCAGACCCCCAATACCAA	502	47.4429	209	21.2045	−1.1618217	1.73 × 10^−24^	**	S	↓
vvi-miR3633a-3p	TTCCTATACCACCCATTCCCTA	1126	106.4158	366	37.1332	−1.5189308	7.32 × 10^−79^	**	S	↓
vvi-miR3633a-5p	GGAATGGATGGTTAGGAGAG	22	2.0792	5	0.5073	−2.0351175	0.0017265	**	S	↓
vvi-miR3633b-3p	GTTCCCATGCCATCCATTCCTA	23	2.1737	5	0.5073	−2.0992418	0.0010695	**	S	↓
vvi-miR3634-3p	TTTCCGACTCGCACTCATGCCGT	84 442	7980.4308	24 524	2488.126	−1.6814071	0	**	S	↓
vvi-miR3635-3p	ATTATGTCCCACACATGCCTC	31	2.9297	20	2.0291	−0.529913	0.2031014		S	
vvi-miR3636-3p	GTCTGTCGGAGAAGCAAGTCGGAG	137	12.9476	53	5.3772	−1.2677577	1.21 × 10^−8^	**	S	↓
vvi-miR3637-3p	TCGACAAGACACAATGCATAAATG	9	0.8506	10	1.0146	0.2543583	0.7015164		D	
vvi-miR3637-5p	TGTATTGTGTTTTGTCGGAAAATA	10	0.9451	15	1.5219	0.6873347	0.2450485		D	
vvi-miR3639-5p	TTGACTTCTGAAAGGCTAAAAGCT	903	85.3406	761	77.2086	−0.1444707	0.0417909		D	
vvi-miR3640-3p	ATCGAAAAGGCATCATCAATCAGG	104	9.8288	163	16.5375	0.750654	2.71 × 10^−5^		S	
vvi-miR3640-5p	ACCTGATTGGTGATGCTTTTTTGG	43	4.0638	28	2.8408	−0.5165321	0.141182		S	
vvi-miR390	AAGCTCAGGAGGGATAGCGCC	60	5.6705	32	3.2466	−0.8045463	0.0097833		S	
vvi-miR393a	TCCAAAGGGATCGCATTGATCC	245	23.1544	66	6.6961	−1.7898934	7.44 × 10^−23^	**	D	↓
vvi-miR393b	TCCAAAGGGATCGCATTGATCC	244	23.0599	66	6.6961	−1.7839933	1.13 × 10^−22^	**	D	↓
vvi-miR394a	TTGGCATTCTGTCCACCTCC	399	37.7086	92	9.334	−2.0143262	3.51 × 10^−42^	**	D	↓
vvi-miR394b	TTGGCATTCTGTCCACCTCC	365	34.4954	78	7.9136	−2.1239979	2.45 × 10^−41^	**	S	↓
vvi-miR394c	TTGGCATTCTGTCCACCTCC	399	37.7086	92	9.334	−2.0143262	3.51 × 10^−42^	**	D	↓
vvi-miR395a	CTGAAGTGTTTGGGGGAACTC	46	4.3474	8	0.8117	−2.4211343	3.15 × 10^−7^	**	S	↓
vvi-miR395b	CTGAAGTGTTTGGGGGAACTC	46	4.3474	8	0.8117	−2.4211343	3.15 × 10^−7^	**	S	↓
vvi-miR395c	CTGAAGTGTTTGGGGGAACTC	46	4.3474	8	0.8117	−2.4211343	3.15 × 10^−7^	**	S	↓
vvi-miR395d	CTGAAGTGTTTGGGGGAACTC	46	4.3474	8	0.8117	−2.4211343	3.15 × 10^−7^	**	S	↓
vvi-miR395e	CTGAAGTGTTTGGGGGAACTC	46	4.3474	8	0.8117	−2.4211343	3.15 × 10^−7^	**	S	↓
vvi-miR395f	CTGAAGTGTTTGGGGGAACTC	46	4.3474	8	0.8117	−2.4211343	3.15 × 10^−7^	**	S	↓
vvi-miR395g	CTGAAGTGTTTGGGGGAACTC	46	4.3474	8	0.8117	−2.4211343	3.15 × 10^−7^	**	S	↓
vvi-miR395h	CTGAAGTGTTTGGGGGAACTC	46	4.3474	8	0.8117	−2.4211343	3.15 × 10^−7^	**	S	↓
vvi-miR395i	CTGAAGTGTTTGGGGGAACTC	46	4.3474	8	0.8117	−2.4211343	3.15 × 10^−7^	**	S	↓
vvi-miR395j	CTGAAGTGTTTGGGGGAACTC	46	4.3474	8	0.8117	−2.4211343	3.15 × 10^−7^	**	S	↓
vvi-miR395k	CTGAAGTGTTTGGGGGAACTC	46	4.3474	8	0.8117	−2.4211343	3.15 × 10^−7^	**	S	↓
vvi-miR395l	CTGAAGTGTTTGGGGGAACTC	46	4.3474	8	0.8117	−2.4211343	3.15 × 10^−7^	**	S	↓
vvi-miR395m	CTGAAGTGTTTGGGGGAACTC	46	4.3474	8	0.8117	−2.4211343	3.15 × 10^−7^	**	S	↓
vvi-miR396a	TTCCACAGCTTTCTTGAACT	177	16.7279	37	3.7539	−2.1557942	3.42 × 10^−21^	**	D	↓
vvi-miR396b	TTCCACAGCTTTCTTGAACTT	2655	250.9183	780	79.1363	−1.6648062	3.95 × 10^−209^	**	D	↓
vvi-miR396c	TTCCACAGCTTTCTTGAACTG	5960	563.2667	1333	135.2419	−2.058276	0	**	S	↓
vvi-miR396d	TTCCACAGCTTTCTTGAACTG	5961	563.3612	1331	135.039	−2.0606841	0	**	S	↓
vvi-miR397a	TCATTGAGTGCAGCGTTGATG	21	1.9847	0	0.01	−7.632777	1.03 × 10^−6^	**	S	↓
vvi-miR398a	TGTGTTCTCAGGTCACCCCTT	15	1.4176	62	6.2903	2.1496783	6.38 × 10^−9^	**	S	↑
vvi-miR398b	TGTGTTCTCAGGTCGCCCCTG	111	10.4904	2	0.2029	−5.692157	2.91 × 10^−29^	**	S	↓
vvi-miR398c	TGTGTTCTCAGGTCGCCCCTG	111	10.4904	2	0.2029	−5.692157	2.91 × 10^−29^	**	S	↓
vvi-miR399g	TGCCAAAGGAGATTTGCCCCT	141	13.3256	71	7.2034	−0.8874506	1.52 × 10^−5^		S	
vvi-miR403a	TTAGATTCACGCACAAACTCG	2230	210.7525	1640	166.3891	−0.3409888	2.89 × 10^−13^		S	
vvi-miR403b	TTAGATTCACGCACAAACTCG	2209	208.7678	1620	164.36	−0.34504	2.04 × 10^−13^		S	
vvi-miR403c	TTAGATTCACGCACAAACTCG	2230	210.7525	1640	166.3891	−0.3409888	2.89 × 10^−13^		S	
vvi-miR403d	TTAGATTCACGCACAAACTCG	2209	208.7678	1620	164.36	−0.34504	2.04 × 10^−13^		S	
vvi-miR403e	TTAGATTCACGCACAAACTCG	2210	208.8623	1621	164.4614	−0.3448031	2.09 × 10^−13^		S	
vvi-miR403f	TTAGATTCACGCACAAACTCG	2231	210.847	1645	166.8964	−0.3372437	4.99 × 10^−13^		S	
vvi-miR408	ATGCACTGCCTCTTCCCTGGC	143	13.5146	8	0.8117	−4.0574284	2.47 × 10^−31^	**	S	↓
vvi-miR477a	TCCCTCAAAGGCTTCCAATTT	382	36.102	150	15.2185	−1.2462526	4.80 × 10^−21^	**	D	↓
vvi-miR477b-3p	CGAAGTCTTTGGGGAGAGTGG	30	2.8352	1	0.1015	−4.8038989	4.38 × 10^−8^	**	S	↓
vvi-miR479	TGTGGTATTGGTTCGGCTCATC	75	7.0881	11	1.116	−2.6670619	4.05 × 10^−12^	**	S	↓
vvi-miR482	TCTTTCCTACTCCTCCCATTCC	12 677	1198.0759	4663	473.093	−1.3405236	0	**	S	↓
vvi-miR535a	TGACAACGAGAGAGAGCACGC	126	11.908	40	4.0583	−1.5529837	1.92 × 10^−10^	**	S	↓
vvi-miR535b	TGACAACGAGAGAGAGCACGC	126	11.908	40	4.0583	−1.5529837	1.92 × 10^−10^	**	S	↓
vvi-miR535c	TGACAACGAGAGAGAGCACGC	126	11.908	40	4.0583	−1.5529837	1.92 × 10^−10^	**	S	↓
vvi-miR828a	TCTTGCTCAAATGAGTATTCCA	17	1.6066	3	0.3044	−2.3999705	0.0025331	**	S	↓

### 3.3. Identification of novel miRNAs in grapevine

The identification of novel miRNAs from the sequence data was carried out using bioinformatics analysis based on novel miRNA annotations criteria as described by Meyers *et al.* [41], in which UNAfold (http://mfold.rna.albany.edu/?q=mfold/RNA-Folding-Form) software was used to predict the secondary structures of pre-miRNAs [42,43], while only those with stable hairpin structures were considered. Based on previous reports, the minimal folding free index (MFEI) was an important feature used to distinguish miRNAs from other noncoding RNAs [44], and miRNAs with MEFI value exceeding 0.80 are anticipated as candidate miRNAs. In the current investigation, a total of 98 novel miRNA candidates were predicted from both control and Cu libraries. As shown in a Venn diagram, 24 novel miRNAs were the common ones in both libraries, while 22 novel miRNAs were only detected in the Cu library and 52 novel miRNAs were specific to the control library (electronic supplementary material, figure S6b). The characteristic of these candidate novel miRNAs could be attributed to the fact that Cu stress can repress or induce the expression of some miRNAs. In addition, most of the novel miRNAs had low expression amounts compared with the known miRNAs, which was consistent with previous reports in *Arabidopsis* [12,45] and wheat [46]. However, several miRNAs, such as novel_mir_42 and novel_mir_43, were present at high expression amounts, which was different from previous results.

### 3.4. Discovery of Cu-responsive miRNAs in grapevine

**Figure 1.**
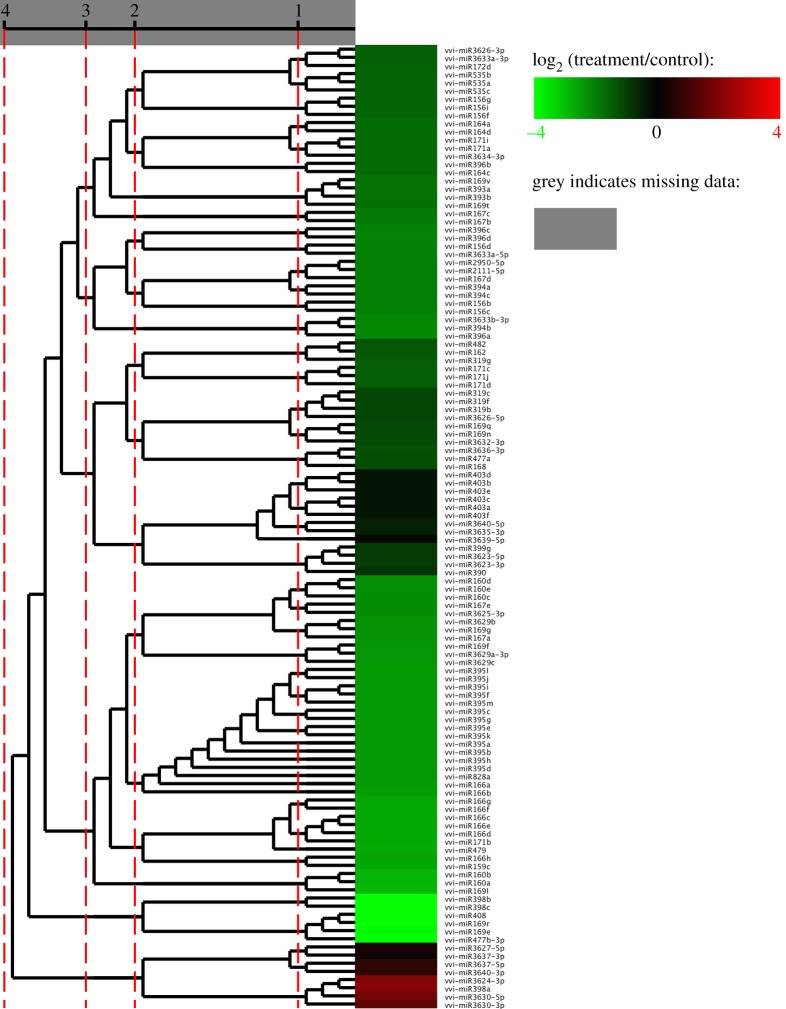
Cluster analysis of known miRNAs expression. The similar expression pattern in known miRNA level was grouped together. Red colours indicated that the expression of miRNA in Cu stress was higher than that of the control. Green colours indicated that the expression of miRNA in Cu stress was lower than that of the control. Grey indicated that there was at least one sample without expression of miRNA.

**Figure 2.**
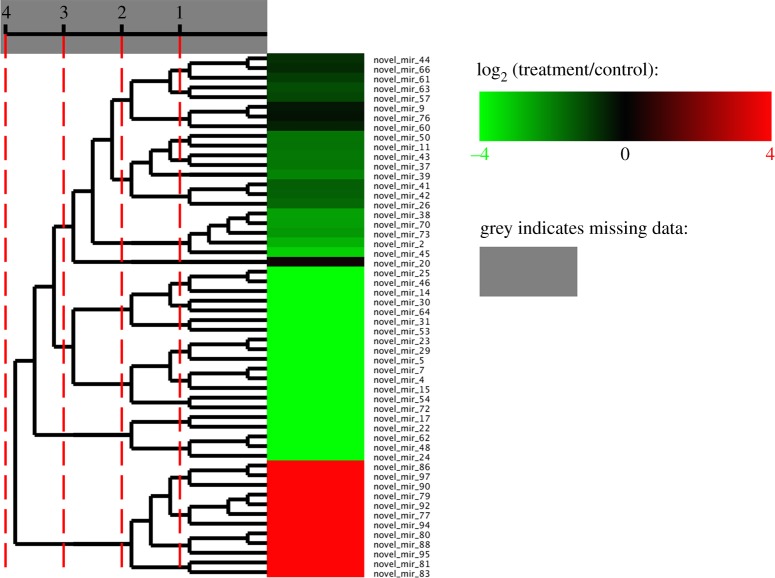
Cluster analysis of novel miRNAs expression. The similar expression pattern in novel miRNA level was grouped together. Red colours indicated that the expression of miRNA in Cu stress was higher than that of the control. Green colours indicated that the expression of miRNA in Cu stress was lower than that of the control. Grey indicated that there was at least one sample without expression of miRNA.

To identify Cu-responsive miRNAs in grapevine, differential expression analysis of 118 known and 54 novel miRNAs were carried out in both control and Cu treatment libraries. In total, 147 miRNAs including 100 known and 47 novel miRNAs were identified as being differentially expressed in response to Cu stress [|log_2_ fold-change (log_2_FC)| ≥ 1, *p*-value < 0.05] (tables 2 and 3). The majority of these Cu-responsive miRNAs were downregulated, except that four known miRNAs (figure 1 and table 2) and 12 novel ones (figure 2 and table 3), showed upregulated patterns (electronic supplementary material, figures S7 and S8). In known miRNAs, vvi-miR398b and vvi-miR398a were found to be the most significantly downregulated and upregulated miRNAs, with fold changes −5.69 and 2.14, respectively. An interesting revelation was that diverse members of the same miRNA family exhibited conspicuous discrepancy in their responses to Cu stress. Similarly, some novel miRNAs including novel_mir_17 and novel_mir_83 were found to be the most significantly downregulated and upregulated miRNAs, with fold changes −15.81 and 16.08, respectively. In addition, 12 novel miRNAs (novel_mir_77, novel_mir_79, novel_mir_80, novel_mir_81, novel_mir_83, novel_mir_86, novel_mir_88, novel_mir_90, novel_mir_92, novel_mir_94, novel_mir_95 and novel_mir_97) were induced by Cu treatment, which only be detected in Cu treated grapevines; whereas, another 20 novel miRNAs (novel_mir_4, novel_mir_5, novel_mir_7, novel_mir_14, novel_mir_15, novel_mir_17, novel_mir_22, novel_mir_23, novel_mir_24, novel_mir_25, novel_mir_29, novel_mir_30, novel_mir_31, novel_mir_46, novel_mir_48, novel_mir_53, novel_mir_54, novel_mir_62, novel_mir_64 and novel_mir_72) were repressed by Cu treatment, and found only in the control but not in Cu treatment (table 3). In general, the significant variations in miRNA expression patterns indicated that miRNAs may play essential roles in response to Cu stress in grapevine.

**Table 3.** The differentially-expressed of novel miRNAs identified from grapevine in respond to Cu stress. -std, standardized expression of miRNAs in sample; **, | log_2_FC| ≥ 1 and *p*-value < 0.01, *, | log_2_FC| ≥ 1 and 0.01 < *p*-value < 0.05, hollow, no obvious difference. ↑,↓ denote upregulated, downregulated.

miR-name	novel_miRNA_sequence	control	CK-std	Cu-treated	Cu-std	fold-change (log_2_ Cu/CK)	*p*-value	sig-lable	regulated
novel_mir_11	TAACTCTGAGTATAAGGCACC	131	12.3805	35	3.551	−1.801772	5.42 × 10^−13^	**	↓
novel_mir_14	TCATGTCTTAATGAATTGCCT	21	1.9847	0	0.01	−7.632777	1.03 × 10^−6^	**	↓
novel_mir_15	TATGGACTTCTCTTAACCCTGT	12	1.1341	0	0.01	−6.825404	0.000384	**	↓
novel_mir_17	ATTCGAACTCAAGACTAAGGT	6104	576.8758	0	0.01	−15.81597	0	**	↓
novel_mir_2	TTGAGAGTGATTTTAGAAAAT	127	12.0025	17	1.7248	−2.798834	1.43 × 10^−20^	**	↓
novel_mir_20	TTCATGGACGTTGATAAGATCCT	6994	660.9878	7626	773.7094	0.2271682	1.79 × 10^−21^		
novel_mir_22	TTCCCAATGCCGCCCATTCCAA	5598	529.0549	0	0.01	−15.69113	0	**	↓
novel_mir_23	AGGCAGTCACCTTGGCTAACT	15	1.4176	0	0.01	−7.147307	5.33 × 10^−5^	**	↓
novel_mir_24	TTCTTGTGATCTTGTTGTTTC	620	58.5949	0	0.01	−12.51656	5.74 × 10^−178^	**	↓
novel_mir_25	TGAACTCCATCCTTTGAATGA	21	1.9847	0	0.01	−7.632777	1.03 × 10^−6^	**	↓
novel_mir_26	TTGGGATTGAGCCTTATGGGC	27	2.5517	8	0.8117	−1.65244	0.0024307	**	↓
novel_mir_29	TTGACATCGATGATATTGAGG	15	1.4176	0	0.01	−7.147307	5.33 × 10^−5^	**	↓
novel_mir_30	TTGTAGGAGCCACAAAATTCTGT	18	1.7011	0	0.01	−7.410324	7.40 × 10^−6^	**	↓
novel_mir_31	TATTCCTTGATATTGCATGTT	27	2.5517	0	0.01	−7.995315	1.98 × 10^−8^	**	↓
novel_mir_37	GTTGGAAGCCGGTGGGGGACC	847	80.0481	218	22.1176	−1.855672	9.22 × 10^−79^	**	↓
novel_mir_38	GTTGGAAGTCGGTGGGGGAAC	209	19.7521	34	3.4495	−2.517547	1.70 × 10^−29^	**	↓
novel_mir_39	GTTGGAAGTCGGTGGGGGACC	373	35.2514	82	8.3195	−2.083112	3.23 × 10^−41^	**	↓
novel_mir_4	CAATCAGCGGCTGAGATAAGC	13	1.2286	0	0.01	−6.940871	0.0001988	**	↓
novel_mir_41	TTCCACGGCTTTCTTGAACTT	2122	200.5456	707	71.7299	−1.483284	5.76 × 10^−142^	**	↓
novel_mir_42	TCTTACCAACACCTCCCATTCC	19 277	1821.8276	6256	634.7136	−1.521209	0	**	↓
novel_mir_43	TTCCCAAGACCCCCCATGCCAA	14 146	1336.9079	3601	365.3459	−1.871565	0	**	↓
novel_mir_44	AGTTACTAATTCATGATGTGGC	20	1.8902	11	1.116	−0.760202	0.1625223		
novel_mir_45	CGGTCCTGGGCCTCTGGCCTT	124	11.719	12	1.2175	−3.266856	3.59 × 10^−23^	**	↓
novel_mir_46	TTCCCACGGCTTTCTTGAACT	21	1.9847	0	0.01	−7.632777	1.03 × 10^−6^	**	↓
novel_mir_48	CTTGAACTCCAATTTGCACCC	117	11.0574	0	0.01	−10.1108	3.67 × 10^−34^	**	↓
novel_mir_5	CTTCTTATTCTTTAAAAGGACT	14	1.3231	0	0.01	−7.047778	0.0001029	**	↓
novel_mir_50	TAACAACTTAAGCTTTTGGGT	23	2.1737	6	0.6087	−1.83635	0.0027137	**	↓
novel_mir_53	AAGGAATTACAAAAGAAATTC	25	2.3627	0	0.01	−7.884293	7.37 × 10^−8^	**	↓
novel_mir_54	TTGCCACTGAGTCTAGAGATG	11	1.0396	0	0.01	−6.699885	0.0007418	**	↓
novel_mir_57	TTACACAGAGAGATGACGGTGG	37	3.4968	16	1.6233	−1.107106	0.0084634	**	↓
novel_mir_60	AGAAGAGAGAGAGTACAGCTA	20	1.8902	13	1.3189	−0.519204	0.3201281		
novel_mir_61	CATTATGTTGATTTCTTTGTT	94	8.8837	45	4.5656	−0.960356	0.000165		
novel_mir_62	TTTTGTTGCTGGTCATCTAGTC	230	21.7368	0	0.01	−11.08592	1.82 × 10^−66^	**	↓
novel_mir_63	TTCTATCGTCATCCTTCCTTG	25	2.3627	10	1.0146	−1.219525	0.0202609	*	↓
novel_mir_64	TTGACTTTGGTGTTTTGGACC	19	1.7956	0	0.01	−7.488322	3.83 × 10^−6^	**	↓
novel_mir_66	TGTGGACATTGTTTCAGGGCT	39	3.6858	23	2.3335	−0.659482	0.0812967		
novel_mir_7	CCCTTTGGAAGTGCTAAGCGCC	13	1.2286	0	0.01	−6.940871	0.0001988	**	↓
novel_mir_70	CATGTGCCCCTCTTCCCCATC	55	5.1979	9	0.9131	−2.509084	1.06 × 10^−8^	**	↓
novel_mir_72	CATCGTCCGAGGCTATGGCGG	11	1.0396	0	0.01	−6.699885	0.0007418	**	↓
novel_mir_73	TGAGATTGAAGACTTTGATGT	326	30.8096	58	5.8845	−2.388388	1.43 × 10^−42^	**	↓
novel_mir_76	ACAAGACTTAGGAAGAATGCACC	1038	98.0991	787	79.8465	−0.297011	1.24 × 10^−5^		
novel_mir_77	CATATCATGCTCTTTAGGACT	0	0.01	14	1.4204	7.1501535	3.55 × 10^−5^	**	↑
novel_mir_79	CTCGTGATGTCAATGATCTCA	0	0.01	14	1.4204	7.1501535	3.55 × 10^−5^	**	↑
novel_mir_80	TTGCCGCACGAGAGATGGCACC	0	0.01	39	3.9568	8.6281903	4.29 × 10^−13^	**	↑
novel_mir_81	TCCCAATGCCGCCCATTCCAA	0	0.01	2343	237.7132	14.536934	0	**	↑
novel_mir_83	CACAAACGACTCTCGGCAACGGA	0	0.01	6829	692.8483	16.080252	0	**	↑
novel_mir_86	TATTCCTTGATATTGCATGTTT	0	0.01	10	1.0146	6.6647673	0.0006565	**	↑
novel_mir_88	AAATTGGCTCTGTAAATTTCT	0	0.01	45	4.5656	8.8346607	5.40 × 10^−15^	**	↑
novel_mir_9	TCGAATCTGTATGAATTGCCT	39	3.6858	28	2.8408	−0.375681	0.2972265		
novel_mir_90	TGAACTGATCTTGATTTTGCAG	0	0.01	10	1.0146	6.6647673	0.0006565	**	↑
novel_mir_92	TGCCAAGAAGCACATTCCTCC	0	0.01	14	1.4204	7.1501535	3.55 × 10^−5^	**	↑
novel_mir_94	TGTAGGGAGTAGAATGCAGCC	0	0.01	16	1.6233	7.3427858	8.26 × 10^−6^	**	↑
novel_mir_95	TTAGATGATCATCAACAAACA	0	0.01	61	6.1889	9.2735392	4.62 × 10^−20^	**	↑
novel_mir_97	TCTGGAAGCAATCAGGAGACT	0	0.01	10	1.0146	6.6647673	0.0006565	**	↑

### 3.5. Prediction of target mRNAs for the known and novel miRNAs in grapevine

To systematically understand the potential biological functions of these identified miRNAs, it was essential to search for their target genes. Following the rules suggested by Allen *et al.* and Schwab *et al.* [11,32], the detailed annotation results of 92 and 51 putative target genes for 79 known and 22 novel miRNAs are shown in electronic supplementary material, tables S1 and S2, respectively. Among the targets, there was the phenomenon that several target genes were predicted to be targeted by one miRNA. For example, *GSVIVT01006275001*, *GSVIVT01007740001*, *GSVIVT01016765001*, *GSVIVT01032467001* and *GSVIVT01033670001* were targeted by vvi-miR828a. On the other hand, one target was usually targeted by more than one miRNA. For example, *GSVIVT01022963001* was predicted to be targeted by novel_mir_11 and novel_mir_81. By contrast, only one target was found for vvi-miR168, vvi-miR169v, vvi-miR2111-5p, vvi-miR3626-3p, vvi-miR3626-5p, vvi-miR482, vvi-miR535a, b, c, vvi-miR477b-3p, vvi-miR482, novel_mir_2, novel_mir_37, novel_mir_41, novel_mir_45, novel_mir_46, novel_mir_54, novel_mir_86, novel_mir_88 and novel_mir_92. Interestingly, several targets were cleaved by pairs of miRNAs, such as vvi-miR393a, and vvi-miR393b, both of which targeted *GSVIVT01010995001*, *GSVIVT01021032001*, *GSVIVT01021910001* and *GSVIVT01033011001*, which suggested that the two miRNAs might cooperate to regulate gene expression.

To understand more details about the roles of miRNAs in response to Cu stress, gene ontology (GO) and kyoto encyclopedia of genes and genomes (KEGG) pathway analysis were performed. GO analysis demonstrated that the 92 putative target genes for 79 known miRNAs were involved in 28 biological processes, while biological process, cellular component and molecular function had 16, 9 and 3 regulated processes, respectively (electronic supplementary material, figure S9); 51 putative target genes for 22 novel miRNAs were involved in 21 biological processes, while biological process, cellular component and molecular function had 11, 6 and 4 regulated processes, respectively (electronic supplementary material, figure S10). In addition, the results of KEGG analysis demonstrated that the putative target genes for differentially-expressed known miRNAs were involved in 17 metabolic pathways, among which plant hormone signal transduction was the main metabolic pathway (electronic supplementary material, table S6). Similarly, putative target genes for differentially-expressed novel miRNAs were playing regulatory roles in 44 metabolic pathways, and the plant–pathogen interaction was the main metabolic pathway (electronic supplementary material, table S7). In this study, several miRNAs (e.g. vvi-miR156, 159, 160, 164 and 167 etc.) were predicted to target diverse gene families of transcription factors (TFs) controlling gene expression related to the plant development, morphology and flowering time. Some of these TFs were squamosa-promoter-binding (SPB) proteins (SPLs), auxin response factors (ARFs), ethylene-responsive transcription factor (RAP2-7), myb domain proteins (MYBs), no apical meristem domain transcription factors (NACs), APETALA2 (AP2), homeobox-leucine zipper proteins (REVOLUTA, ATHB-15, and HOX32,), and TCP2-like factor. Besides, a few miRNA targets identified in this study might be involved in biotic and abiotic stresses, such as putative disease resistance RPP13-like protein 1, L-ascorbate oxidase (ASO), polyphenol oxidase (PPO), and Laccase (LAC).

### 3.6. qRT-PCR verification

**Figure 3.**
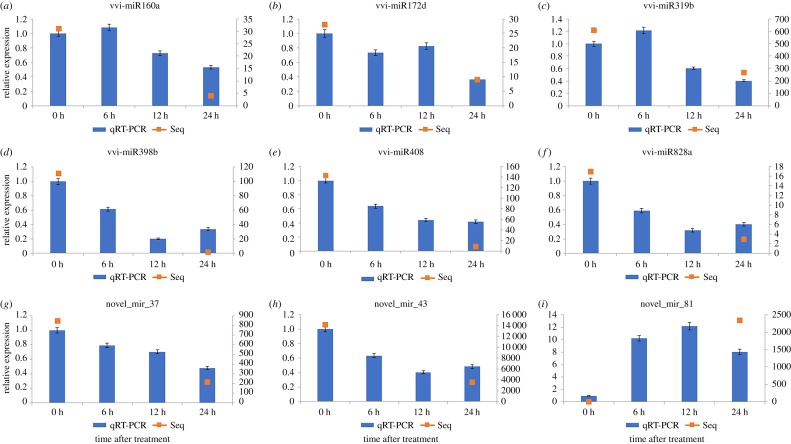
qRT-PCR validations of Cu-responsive known and novel vvi-miRNAs in grapevine. Reference gene was *5.8S rRNA*. The known and novel miRNAs levels in control were arbitrarily set to 1. Each PCR reaction was repeated three times and standard error was shown with bars in the diagram.

To examine the miRNAs expression amounts and verify the sequencing result, nine Cu-responsive miRNAs, including six known miRNAs and three novel miRNAs, were randomly selected for further analysis. Following previous studies [21,47,48], qRT-PCR was carried out to verify the high-throughput sequencing result and investigate the expression patterns of miRNAs under different treatment time periods (0, 6, 12 and 24 h). The findings revealed a moderate consistency with the expression profiles of Cu-responsive miRNAs between high-throughput sequencing and qRT-PCR assays. As shown in figure 3, there exist some differences in expression levels of six known miRNAs varied in different treatment time periods. Overall, the expression profiles of six known miRNAs showed a declining trend, while the expression levels of vvi-miR160a and vvi-miR319b were slightly upregulated at 6 h and significantly downregulated at 12 h in the Cu treatment. The expression levels of vvi-miR172d were slightly higher in the Cu treatment at 12 h and sharply downregulated at 24 h. For the novel miRNAs, the expression profiles of novel_mir_37 were equally declining; the expression profiles of novel_mir_43 were equally declining in the Cu treatment at 6 h and 12 h, while slightly upregulated at 24 h. The expression amounts of novel_mir_81 were sharply upregulated at 6 h and up to peak at 12 h in the Cu treatment, and then slightly downregulated at 24 h.

**Figure 4.**
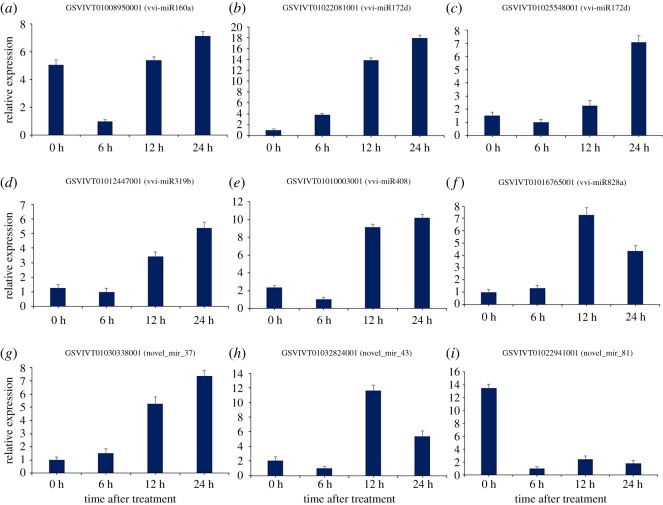
qRT-PCR validations of target genes for differentially expressed vvi-miRNAs in grapevine. The reference gene was *Actin*. The target genes with lower expression amounts were arbitrarily set to 1. Each PCR reaction was repeated three times and standard error was pointed with bars in the diagram.

Meanwhile, the expression profiles of nine target genes (*GSVIVT01008950001*, *GSVIVT01022081001*, *GSVIVT01025548001*, *GSVIVT01012447001*, *GSVIVT01010003001*, *GSVIVT01016765001*, *GSVIVT01030338001*, *GSVIVT0103282400*1 and *GSVIVT01022941001*) cleaved by vvi-miR160a, vvi-miR172d, vvi-miR319b, vvi-miR408, vvi-miR828a, novel_mir_37, novel_mir_43 and novel_mir_81 were also verified by qRT-PCR. As shown in figure 4, the expression amounts of target genes cleaved by vvi-miR172d, vvi-miR319b, vvi-miR408, and novel_mir_37 were increasing, and finally peaked at 48 h in the Cu treatment. Furthermore, the expression amounts of the target genes cleaved by vvi-miR828a and novel_mir_43 were both sharply upregulated at 12 h in the Cu treatment, followed by a decrease. Similarly, the expression amounts of target gene of vvi-miR160a were sharply declining at 6 h and constantly increasing at 12 and 24 h. At 6 h, the expression amounts of target genes of novel_mir_81 were significantly declining, followed by a slight increase at 12 and 24 h.

### 3.7. Experimental verification of miRNA-guided cleavage of target genes using 5′-RLM-RACE

**Figure 5.**
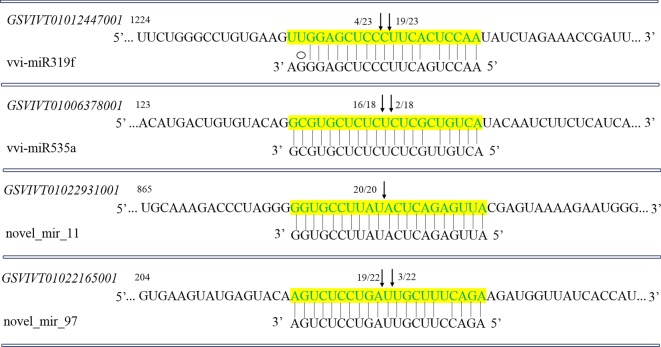
Verification of target genes for vvi-miRNAs by 5′-RLM-RACE. Each top strand (green) depicts a miRNA-complementary site in the target mRNA, and each bottom strand depicts the miRNA (black). Watson–Crick pairing (vertical dashes) and guanine–uracil wobble pairing (ellipses) are indicated. The arrows indicate the 5′ termini of mRNA fragments isolated from grapevine, as identified by cloned RLM-RACE products, with the frequency of clones shown. Only the cloned sequences that matched the correct gene and had 5′ ends within a 100 nt window centred on the miRNA validation are included. The partial mRNA sequences from the target genes were aligned with the miRNAs. The numbers indicate the fraction of cloned PCR products terminating at different positions.

Slicing of target mRNAs can facilitate successful verification of target genes through the method of detecting cleavage products and sites of target mRNAs. To verify the nature of potential miRNA targets and to study the miRNAs regulation on their target genes, a 5′-RLM-RACE experiment was set up and used to map the cleavage sites in four predicted miRNA target genes. The findings demonstrated that the cleavage sites of these four miRNA target genes *GSVIVT01012447001*, *GSVIVT01006378001*, *GSVIVT01022931001* and *GSVIVT01022165001* for vvi-miR319f, vvi-miR535a, novel_mir_11, and novel_mir_97 in this study were at the nucleotide that pairs with the 10th and/or 11th nucleotide of the 5′-end of corresponding miRNAs (figure 5), consistent with previous related reports [8,17,19,20,49]. Moreover, the *GSVIVT01012447001*, *GSVIVT01006378001*, *GSVIVT01022931001* and *GSVIVT01022165001* were confirmed as the true targets of vvi-miR319f, vvi-miR535a, novel_mir_11 and novel_mir_97, respectively. The primers of the 5′-RLM-RACE experiment are as listed in electronic supplementary material, table S5.

### 3.8. Network analysis

**Figure 6.**
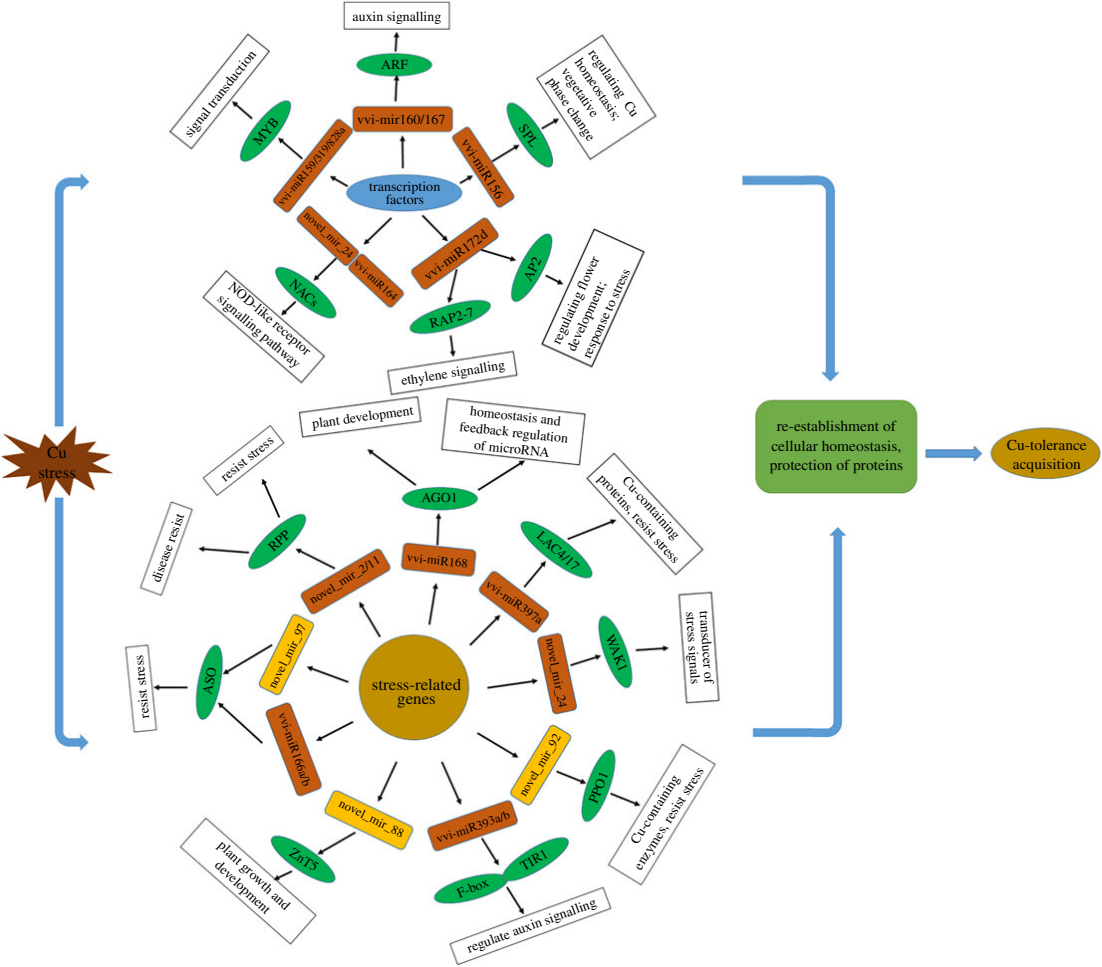
A schematic model for Cu-stress-responsive miRNAs and targets in grapevine. Brown boxes indicate downregulated miRNAs, yellow boxes indicate upregulated miRNAs and green ellipse boxes indicate the target genes.

The network between miRNAs and target genes was elucidated according to GO analysis (figure 6). As shown in figure 6, several transcription factors were found in the target genes, including RAP2-7 (the putative target of vvi-miR172d), SPL (vvi-miR156b, c, d, f, g, i), ARF (vvi-miR160a, b, c, d, e and vvi-miR167a, b, c, d, e), AP2 (vvi-miR172d), NAC (novel_mir_24 and vvi-miR164a, c, d), and MYB (vvi-miR159c, vvi-miR828a and vvi-miR319b, c, f, g), which were involved in plant development and hormone signal transduction, as well as stress resistance. Furthermore, the decreased vvi-miR168, vvi-miR397a, vvi-miR393a, vvi-miR166a, b, novel_mir_2, novel_mir_11, novel_mir_22 and increasing novel_mir_88, novel_mir_92, novel_mir_97 targeted some stress-related genes involved in plant development and regulation of genes encoding anti-stress proteins. Of the target genes examined, *RPP*, *ASO*, *PPO1*, *AGO1*, *LAC4/17*, *ZnT5*, *TIR1* and *ABF* might be the most important genes in the entire network.

## 4. Discussion

MiRNAs, a class of non-coding small RNAs, are important regulators involved in plant growth, development and stress responses that have received increasing attention [17,50–52]. In recent years, a new approach for discovering miRNAs, high-throughput sequencing technology, has been widely used to identify conserved and novel miRNAs in plants, which has enlarged the realm of miRNA research and made miRNA a hotspot of epigenetic research. Abiotic stress, a common stress that seriously affects plant production, has also received increasing attention. The modern research on the use of high-throughput sequencing and miRNA regulation theory have opened up a novel subject in abiotic stress research, including drought [53,54], salt [55,56], waterlogging [57], and heavy metal stresses [58–60]. Cu is one of the essential micronutrients required for healthy growth and development of perennial plants, while its deficiency may cause stunted plant growth, abnormal leaf pigmentation and development; whereas, excessive uses are toxic to plants regardless of their dose. In China, due to long-term waste-water irrigation, over-use of fungicides and pesticides containing Cu in viticulture, Cu stress has become one of the serious environmental crises that seriously affects grapevine growth and development. However, to our knowledge, few such studies have been reported on grapevine. Therefore, the current investigation will help to reveal the molecular mechanisms of the miRNAs in response to Cu stress. More importantly, the study will provide a theoretical foundation for breeding Cu-tolerant grapevine cultivars.

### 4.1. Known and novel miRNAs in grapevine

In our study, some miRNAs, such as vvi-miR164b, vvi-miR159a, b, vvi-miR156a, e, h, vvi-miR3628, vvi-miR3631a, b, c, d, and vvi-miR3638, deposited in miRBase, were not detected, perhaps because these miRNAs have specific expression patterns. The comparison of our dataset from this work with the similar work of vvi-miRNAs from Wang *et al.* [21] revealed that most of the vvi-miRNAs could be observed since the same grapevine cultivar was used in both studies, while only a few members, such as vvi-miR156e, vvi-miR159a, b, vvi-miR160f, vvi-miR535d, e and vvi-miR845a,b,c, could be discovered in one of both studies, which may be derived from the differences in the tissues of grapevine materials used in both studies or Cu induced/depressed miRNAs in this work. Additionally, 98 candidate novel miRNAs were predicted in the two libraries after removing redundant sequences. In contrast to known miRNAs, most novel miRNAs exhibited low expression amounts, consistent with the previous reports in *Arabidopsis* [11,12,45], wheat [46]. However, several miRNAs, such as novel_mir_42 and novel_mir_43, were present in high amounts, which was different from previous results. In the current study, a total of 158 known and 98 novel miRNAs were firstly discovered to be associated with the Cu response.

### 4.2. Differentially expressed miRNAs in grapevine

The abundance of miRNAs can be regarded as an index for estimating the expression amounts of miRNAs. Thus, to identify Cu-responsive miRNAs, the expression amounts of miRNAs between the two libraries were compared. The results showed 147 miRNAs, including 100 known and 47 novel miRNAs, were differentially expressed between the two libraries. Among these, 16 miRNAs (4 known and 12 novel) were significantly upregulated patterns, indicating that these miRNAs might play indispensable roles in response to Cu stress; whereas, 131 miRNAs (96 known and 35 novel) were significantly downregulated, suggesting that Cu-stress treatment suppressed the miRNAs expression level. It is well documented that few Cu-responsive miRNAs, for example, miR398 and miR408, were conserved in various plant species, e.g. *A. thaliana* and Chinese cabbage, and both were found to be negatively regulated by Cu [61,62]. In the current study, vvi-miR398 (vvi-miR398b, and vvi-miR398c) and vvi-miR408 were both found to be negatively regulated by Cu stress, indicating that some miRNAs involved in Cu stress showed consistency among several plant species. However, vvi-miR398a was found to be upregulated by Cu stress, in disagreement with previous reports in *A. thaliana* [62]. It was possible that vvi-miR398a showed diverse induction levels due to the use of the different materials, or certain miRNAs may respond differently to various treatment times of Cu stress.

To further know the molecular function of grapevine miRNAs in response to abiotic stress, the expression patterns of miRNAs between Cu-stress and cold-stress conditions were compared in *Vitis vinifera*. Significant differences were found between Cu and cold stress (electronic supplementary material, table S8). In general, the number of differentially expressed miRNAs after Cu stress is significantly more than after cold stress. Additionally, 39 Cu-inducible miRNAs were found in our libraries, but not induced in cold-stress condition, indicating that these miRNAs possessed Cu-stress-specific properties. The vvi-miR3640 were found to respond to cold stress in *Vitis vinifera*; however, no visible expression change was found under the Cu-stress condition in our experiment, suggesting that it is probably cold-stress-specific miRNA. A previously identified cold-responsive miRNA, vvi-miR3633b in *Vitis vinifera* [63], was not detected in our two libraries. One interpretation of these differentials is that the abundance of vvi-miR3633b in grapevine leaves is too low, so we could not recognize it in the present experimental system. In this study, vvi-miR3636 and vvi-miR3634 were downregulated after Cu stress, however, they showed upregulated expression after cold stress, suggesting that they play different roles in abiotic stress.

### 4.3. Target genes for identified miRNAs in grapevine

Given that miRNAs-mediated post-transcriptional gene regulation by translational repression or endonucleolytic cleavage, the underlying documentation of target prediction is essential to gain insight into the regulatory functions of miRNAs. In this study, a large number of potential targets for known and novel miRNAs were predicated (electronic supplementary material, tables S1 and S2). *SPL7* was targeted by vvi-miR156 in this study, maybe involved in regulating Cu homeostasis in grapevine. Consistent with the previous report of Floyd & Bowman [64], some of the predicated targets in this study were plant-specific transcription factors, such as the AP2, SBP, NAC and ARF families. Among these transcription factors, AP2 and SPB were the important genes known for regulating flower development [65,66]. Additionally, AP2 proteins, one of the most important families of transcriptional regulators, play a crucial role in response to biotic and abiotic stressors [67]. *AtSPL7* can bind directly to CuREs in the miR398 promoter *in vitro* and it appears to be a regulator for Cu homeostasis in *Arabidopsis* [68]. *SPL7* was targeted by vvi-miR156 in this study, maybe involved in regulating Cu homeostasis in grapevine.

In addition to targeting transcription factors, a few targets predicted in this study may also be involved in biotic and abiotic stresses, such as AGO1, RPP13, ASO, PPO, and LAC. Previous works have described the feedback regulation of AGO1 mRNA through the action of miR168 as being a crucial step for proper plant development and regulation of gene expression [69,70]. Additionally, it is reported that several AGO proteins have been shown to be regulated by biotic and abiotic stresses [71,72]. In our study, *VvAGO1* was targeted by vvi-miR168, and involved in resisting Cu stress. The *Arabidopsis* gene families of MIR397, with two members (miR397a and miR397b), have been shown to regulate a subset of the laccase gene family (*AtLAC*) [45,62,73]. Like *CSDs*, *LACs*, which are Cu-containing proteins, play a critical role in Cu-tolerance in plants. Among the predicted members of the miR397 family in grapevine, we only detected vvi-miR397a, which targeted *VvLAC4* and *VvLAC17*, suggesting that vvi-miR397a might play a more important role in response to Cu stress in grapevine than vvi-miR397b. Receptor-like kinases play important roles in defence responses. When plants suffer from abiotic stress, receptor-like kinases may be the first sensor or transducer of stress signals. In the current study, a wall-associated receptor kinase-like 1 (WAK1) targeted by novel_mir_24 was also identified, indicating that novel_mir_24 was an important regulator of the Cu response.

### 4.4. miRNA-guided regulation networks in grapevine of Cu response

To elucidate the networks between miRNAs and their target genes in grapevine, the targets for known and novel miRNAs response to Cu stress were predicted. As expected, several targets belonged to transcription factors such as *SPLs*, *ARFs*, *MYBs*, *NACs* and *RAP2-7*. The majority of these transcription factors were known to play critical roles in plant development and to be responsive to biotic and abiotic stresses (figure 6). The vvi-miR156 was repressed under Cu stress, and its corresponding target genes were triggered, maybe acting as a regulator for Cu homeostasis to adapt to the Cu stress. Moreover, some other predicted miRNA targets in grapevine were potentially involved in hormone signalling, disease resistance, biological metabolism and diverse environmental stresses. For instance, vvi-miR172d targets a transcript-encoding ethylene-responsive transcription factor RAP2-7. It was reported that ethylene may be involved in the regulation of multiple physiological properties by acting as a signal molecule [74]. The vvi-miR160 and vvi-miR167 were repressed under Cu stress, and its corresponding target *ARF* was triggered, maybe involved in the resistance of abiotic stress by acting as a signal molecule. The functional characterization of miR393 as a conserved family has been done in many plants, and four *F-box* genes, *TIR1*, *AFB1*, *AFB2*, and *AFB3*, have been identified and validated as targets of miR393 [61,75,76]. The TIR1/AFBs regulate auxin signalling by proteolysis of auxin/indole-3-acetic acids (Aux/IAA) repressors, and by releasing the activities of auxin response factors (ARFs) [77–79]. As shown in figure 6, vvi-miR393a, b was repressed under Cu stress, and its corresponding target *TIR1* and *AUXIN SIGNALING F-BOX 2* (*AFB2*) were triggered, involved in auxin response and plant development. Likewise, LACs, PPOs and CSDs are Cu-containing enzymes, can oxidize mono- or dihydroxy phenols to quinines and are involved in plant resistance to biotic and abiotic stresses [80]. Of the novel miRNAs, novel_mir_92 increased in Cu stress, and targeted *PPO1*, which may play an important role in Cu stress.

## 5. Conclusion

In summary, in the present study, 158 known and 98 novel miRNAs were identified using high-throughput sequencing; among which, 100 known and 47 novel miRNAs were identified as differentially expressed under Cu stress. The information fills the gaps in the knowledge of grapevine Cu-responsive miRNAs and increases the knowledge of the response of miRNAs to stress. The targets prediction of miRNAs indicates that miRNA may regulate some transcription factors, including AP2, SBP, NAC, MYB and ARF during Cu stress. Subsequently, the expression patterns of nine Cu-responsive miRNAs were validated by qRT-PCR. There existed some consistency in expression levels of Cu-responsive miRNAs between high throughput sequencing and qRT-PCR assays. The target genes for two known and two novel miRNAs showed specific cleavage sites at the 10th and/or 11th nucleotide from the 5′-end of the miRNA corresponding to their miRNA complementary sequences. Our results indicated that a set of miRNAs are Cu-responsive miRNAs and play an important role in Cu stress response. The findings will lay the foundation for exploring the role of the regulation of miRNAs in response to Cu stress and provide valuable gene information for breeding some Cu-tolerant grapevine cultivars.

## Supplementary Material

Figure S1

## Supplementary Material

Figure S2

## Supplementary Material

Figure S3

## Supplementary Material

Figure S4

## Supplementary Material

Figure S5

## Supplementary Material

Figure S6

## Supplementary Material

Figure S7

## Supplementary Material

Figure S8

## Supplementary Material

Figure S9

## Supplementary Material

Figure S10

## Supplementary Material

Table S1

## Supplementary Material

Table S2

## Supplementary Material

Table S3

## Supplementary Material

Table S4

## Supplementary Material

Table S5

## Supplementary Material

Table S6

## Supplementary Material

Table S7

## Supplementary Material

Table S8

## Data accessibility

The sequencing data as part of this study have been uploaded to the NCBI, GEO databases (accession number: GSE78133).
