# Supplementary material for: Identification of copper (Cu) stress-responsive grapevine microRNAs and their target genes by high-throughput sequencing
Source: R Soc Open Sci. 2019 Jan 23;6(1):180735. doi: 10.1098/rsos.180735 (PMC6366190; doi:10.1098/rsos.180735)
Supplement: Figure S1 [file rsos180735supp1.pdf]

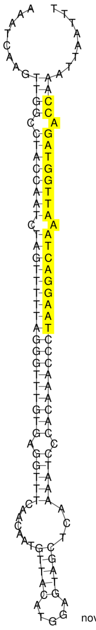

novel_mir_1

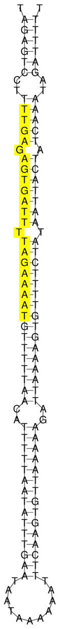

novel_mir_2

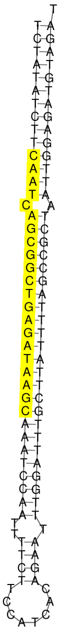

novel_mir_4

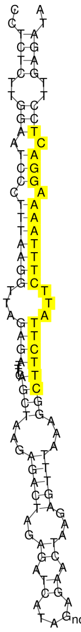

novel_mir_5

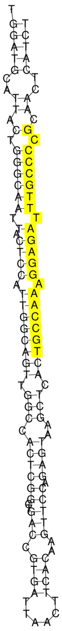

novel_mir_6

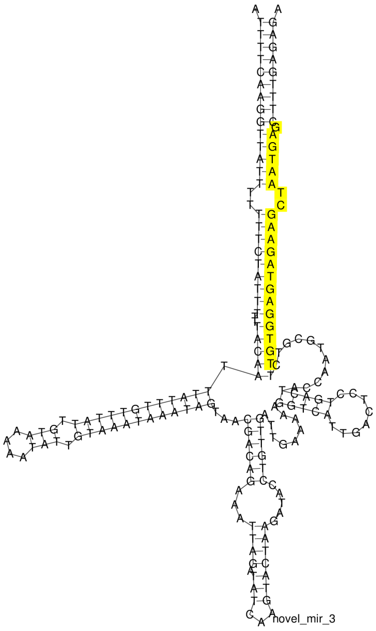

novel_mir_3

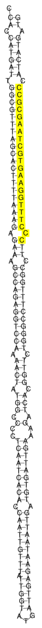

novel_mir_7

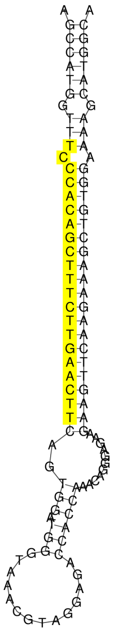

novel_mir_8

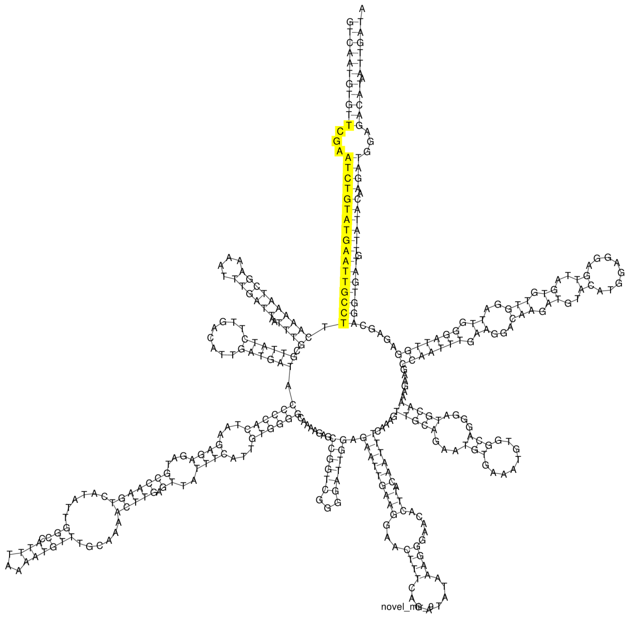

novel_mir_9

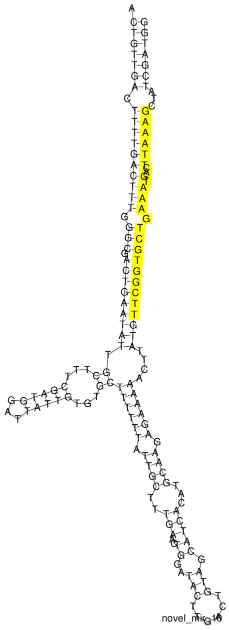

novel_mir_10

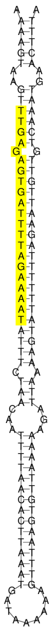

novel_mir_2

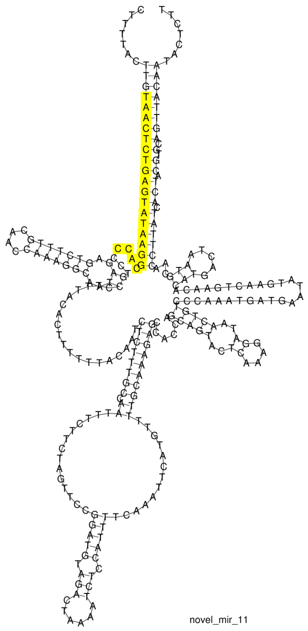

novel_mir_11

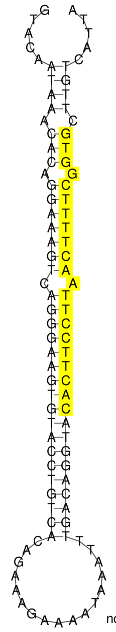
novel_mir_12

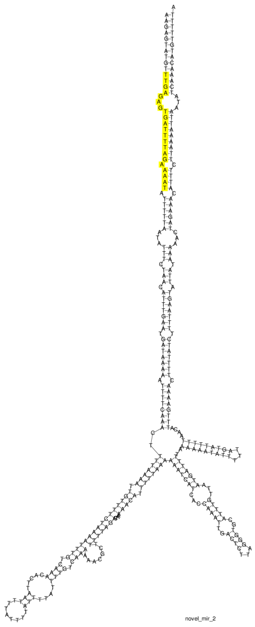
novel_mir_2

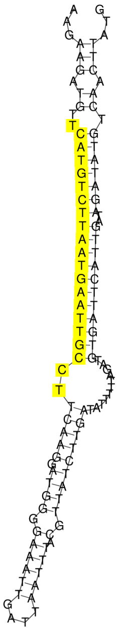
novel_mir_14

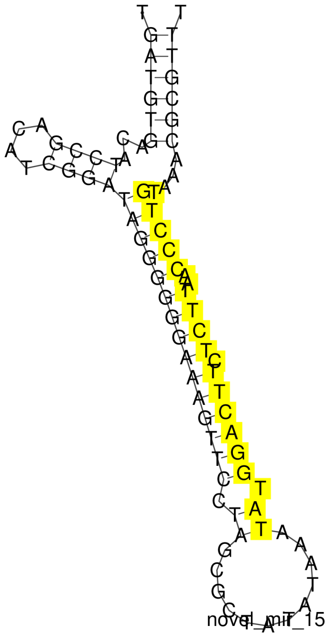
novel_mir_15

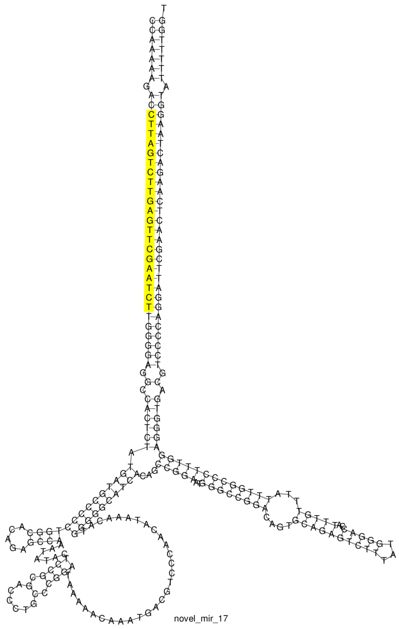
novel_mir_17

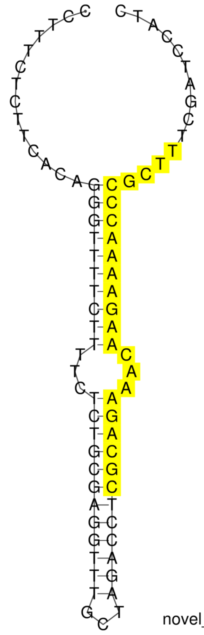
novel_mir_19

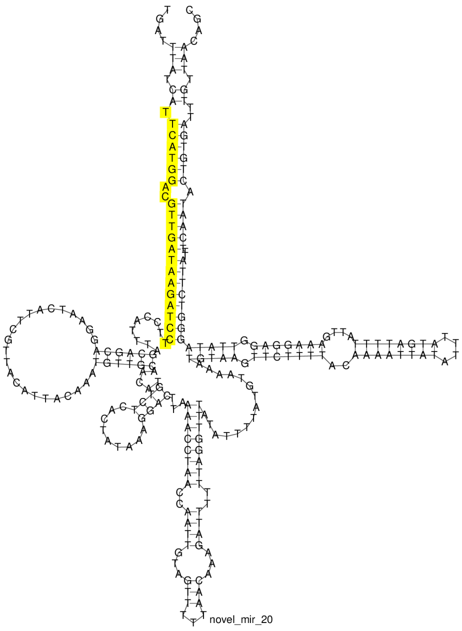

novel_mir_20

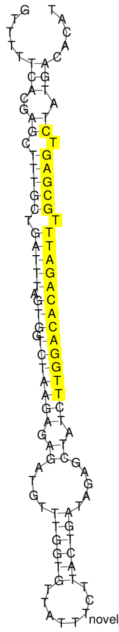

novel_mir_21

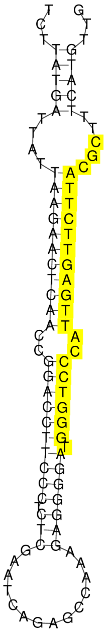

novel_mir_13

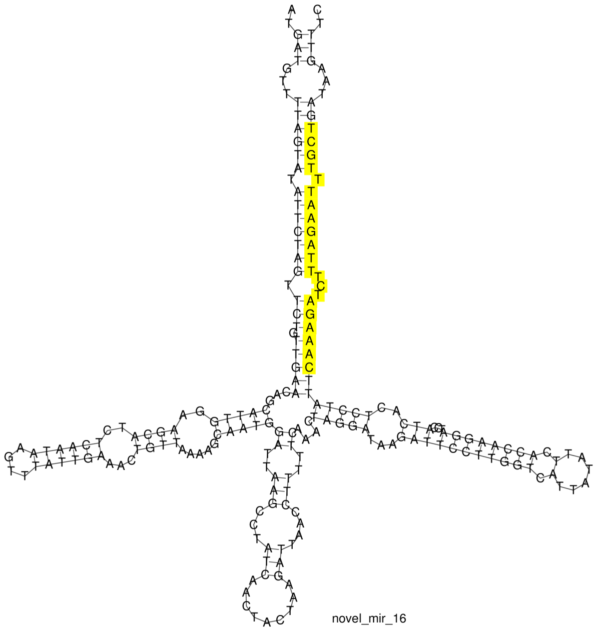

novel_mir_16

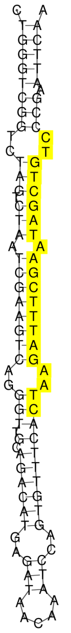

novel_mir_18

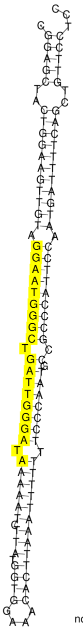

novel_mir_22

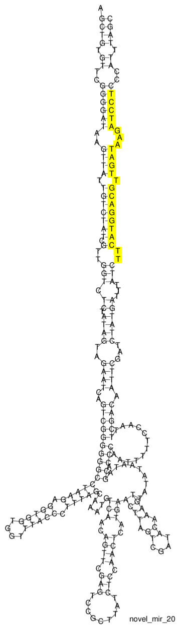
novel_mir_20

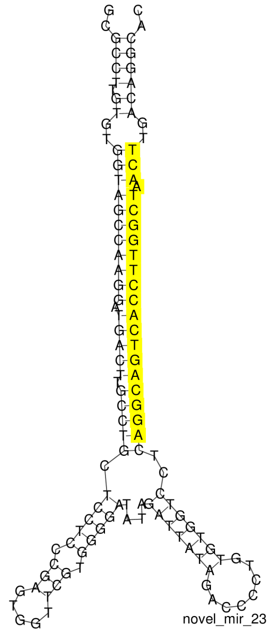
novel_mir_23

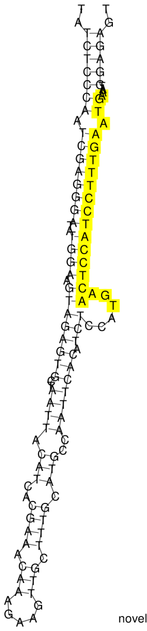
novel_mir_25

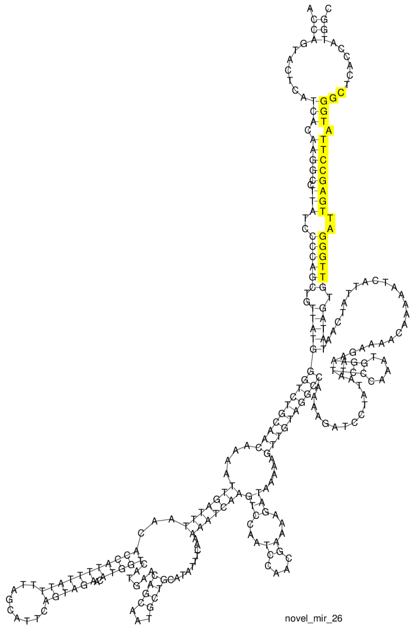
novel_mir_26

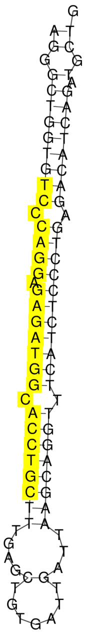
novel_mir_28

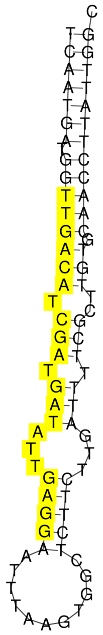
novel_mir_29

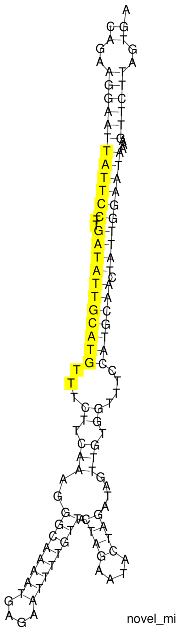

novel_mir_31

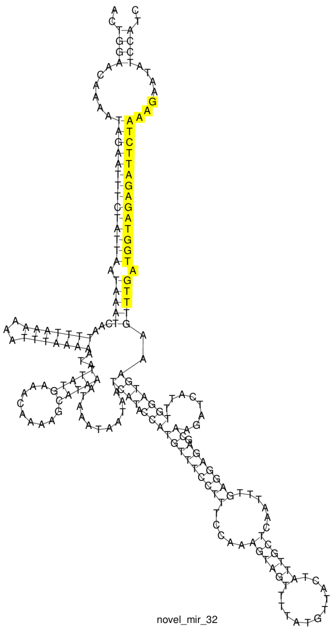

novel_mir_32

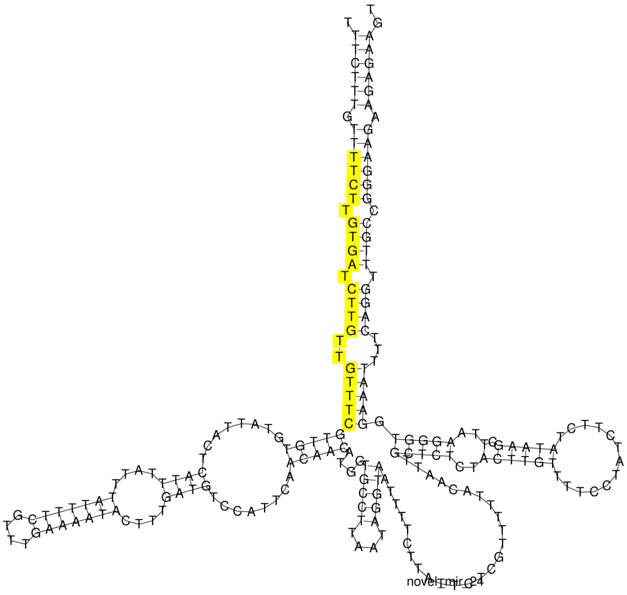

novel_mir_24

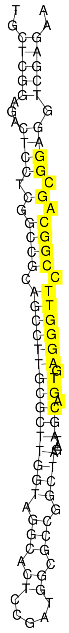

novel_mir_27

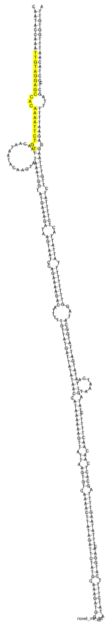

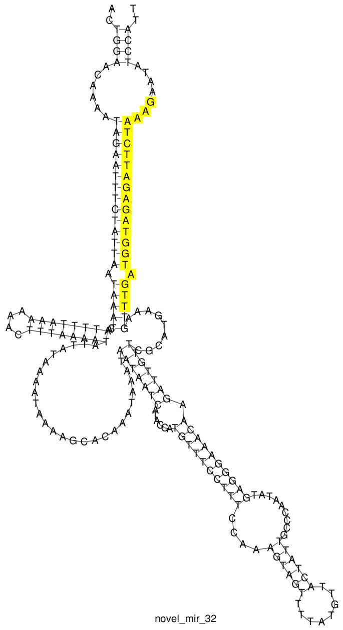

novel_mir_32

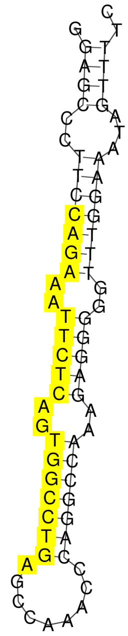

novel_mir_33

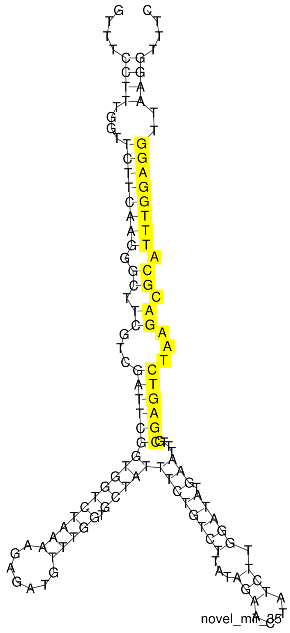

novel_mir_35

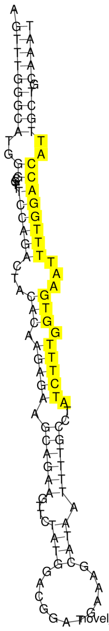

novel_mir_36

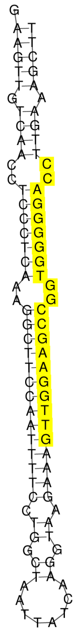

novel_mir_37

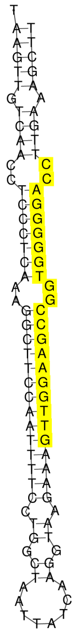

novel_mir_37

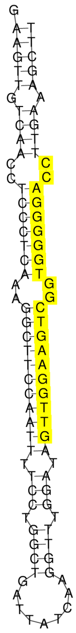

novel_mir_39

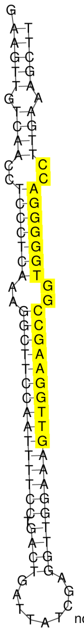

novel_mir_37

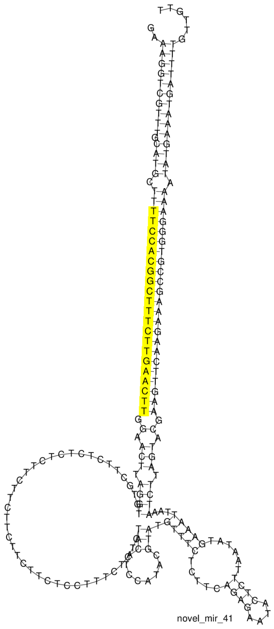

novel_mir_41

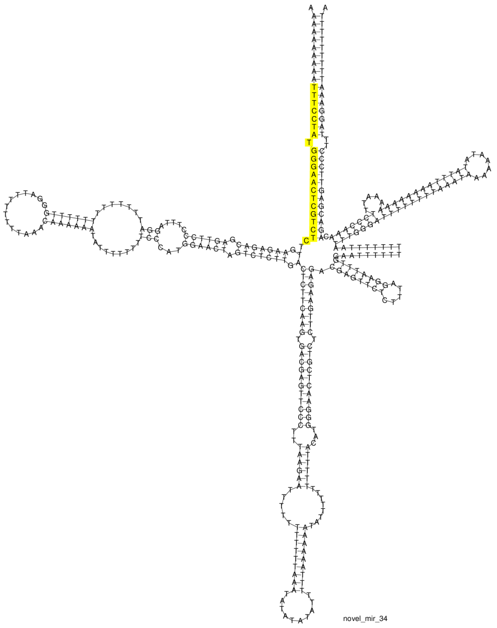

novel_mir_34

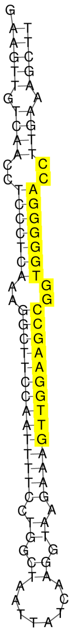

novel_mir_37

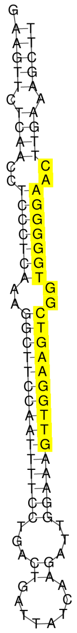

novel_mir_38

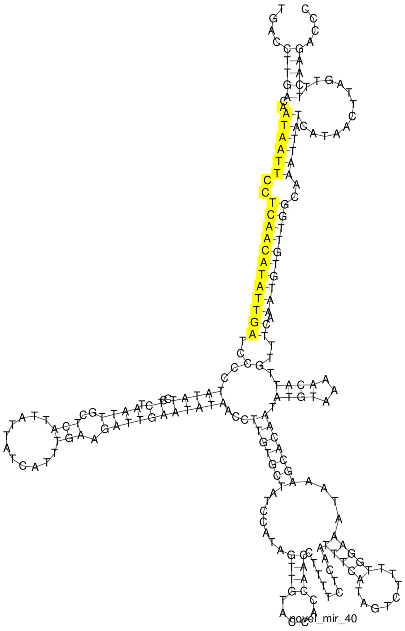

novel_mir_40

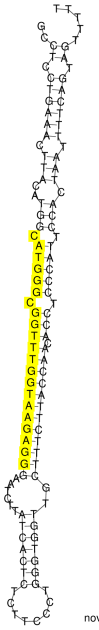
novel_mir_42

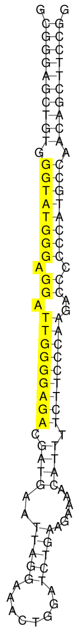
novel_mir_43

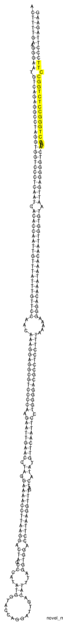
novel_mir_45

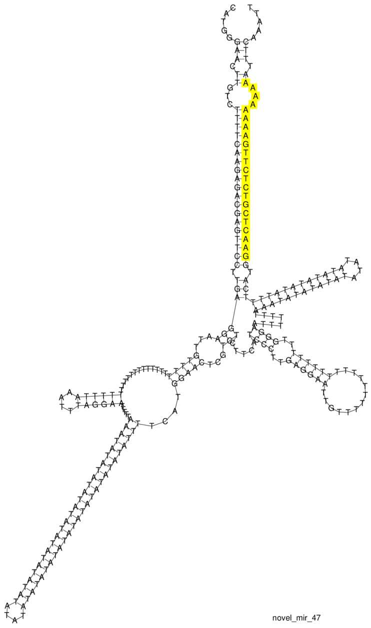
novel_mir_47

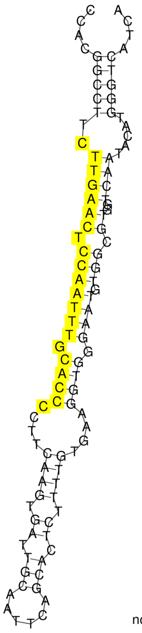
novel_mir_48

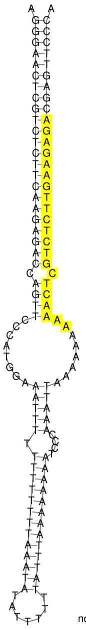
novel_mir_49

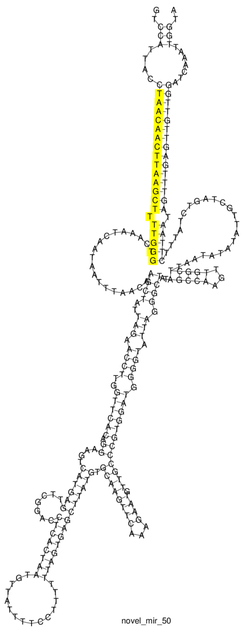

novel_mir_50

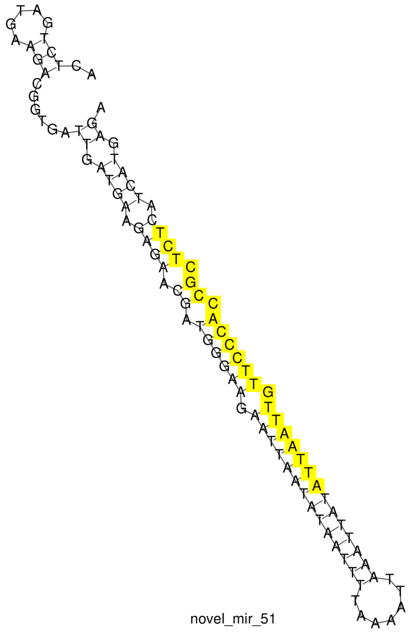

novel_mir_51

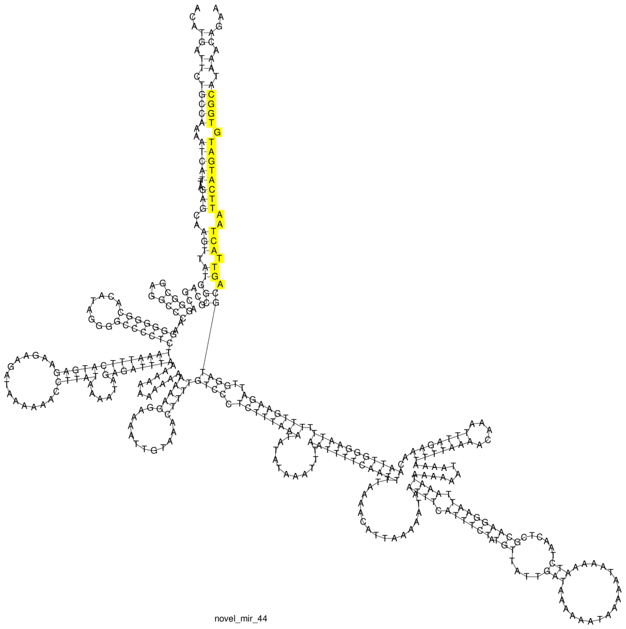

novel_mir_44

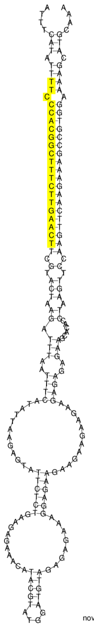

novel_mir_46

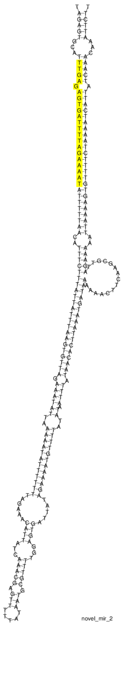

novel_mir_2

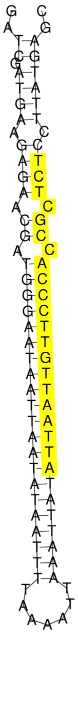

novel_mir_51

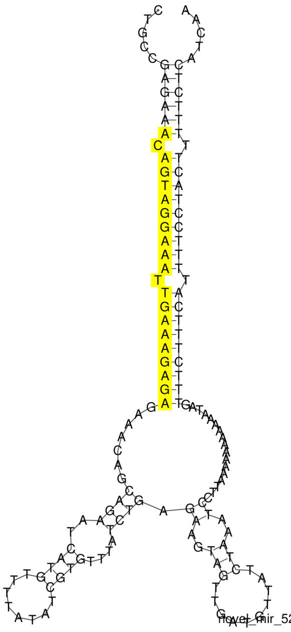

novel_mir_52

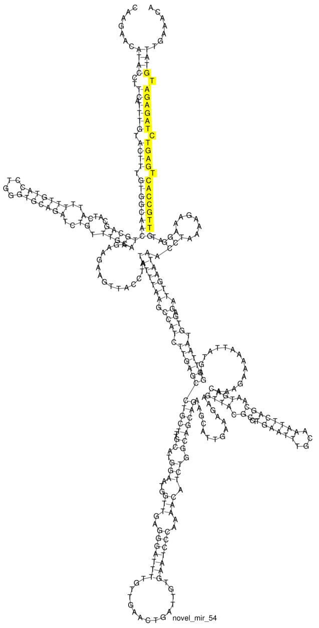

novel_mir_54

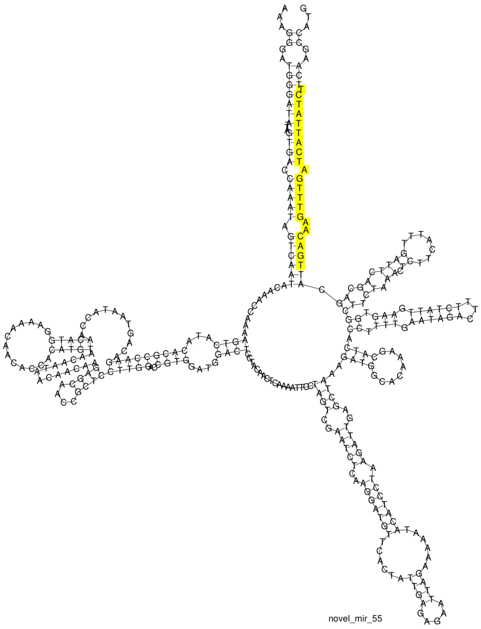

novel_mir_55

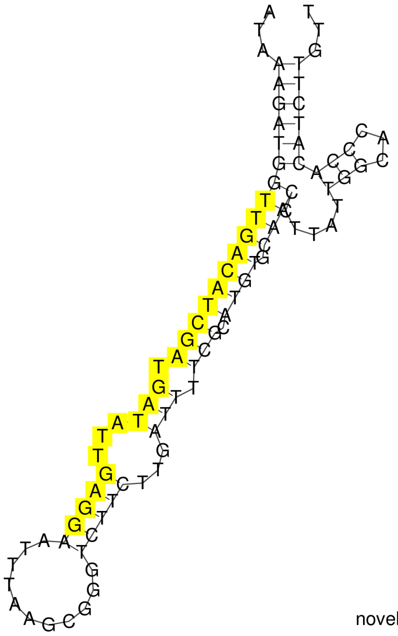

novel_mir_29

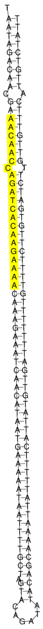

novel_mir_24

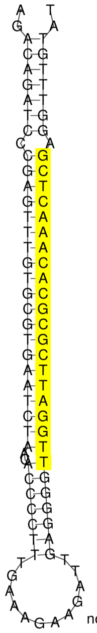

novel_mir_56

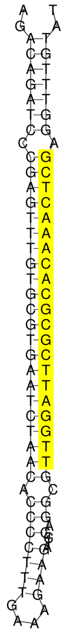

novel_mir_56

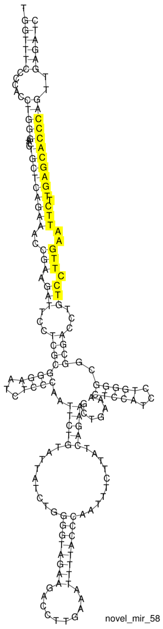

novel_mir_58

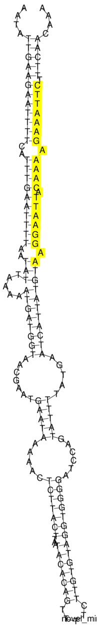

novel_mir_53

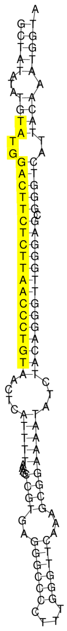

novel_mir_15

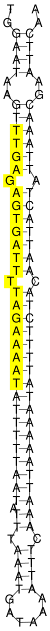

novel_mir_2

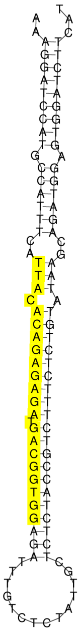

novel_mir_57

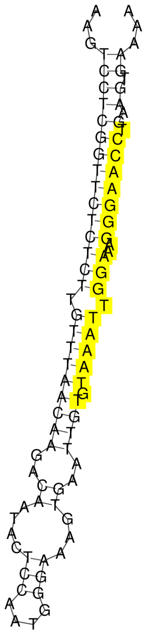

novel_mir_59

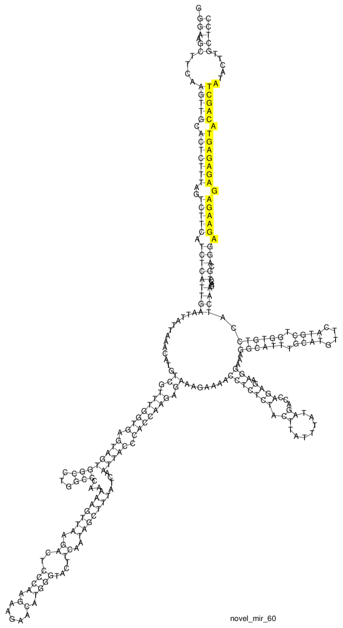

novel_mir_60

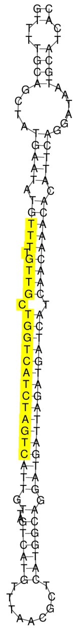

novel_mir_62

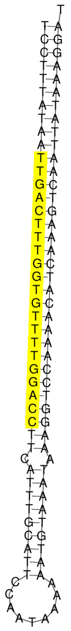

novel_mir_64

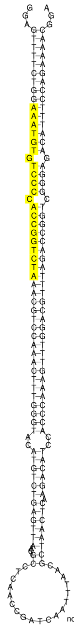

novel_mir_65

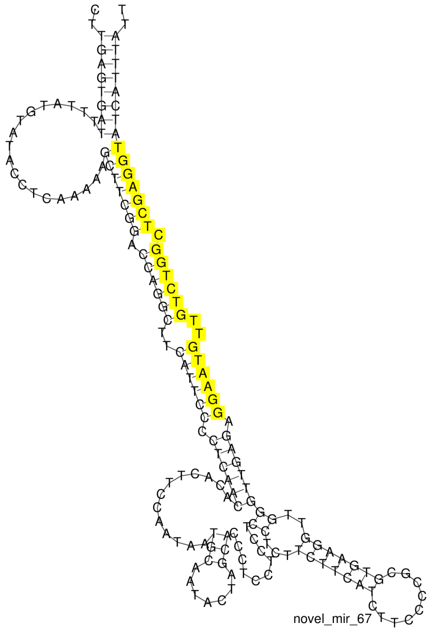

novel_mir_67

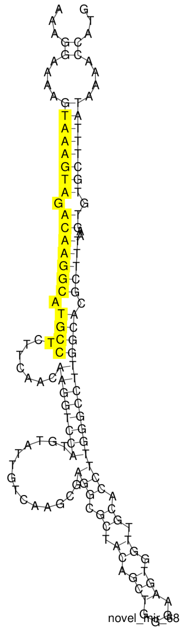

novel_mir_68

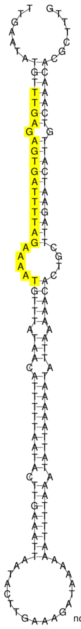

novel_mir_2

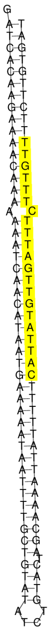

novel_mir_61

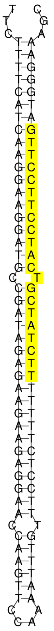

novel_mir_63

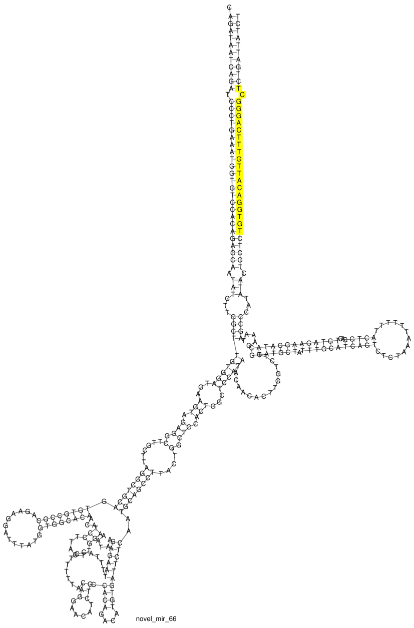

novel_mir_66

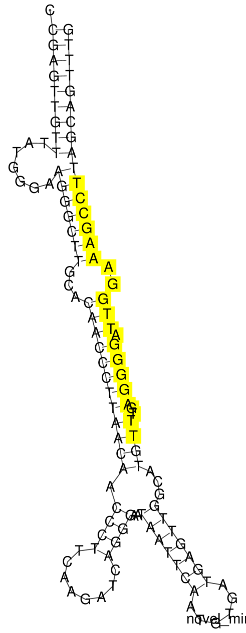

novel_mir_69

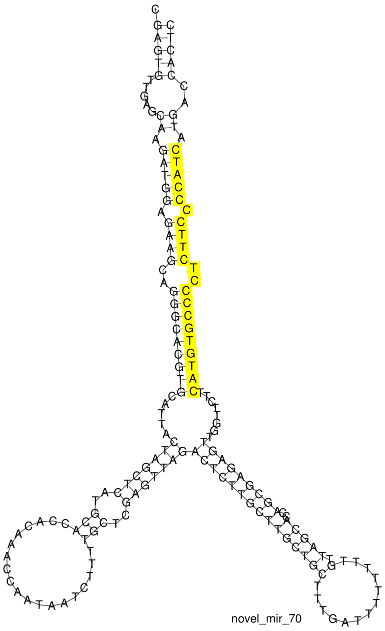

novel_mir_70

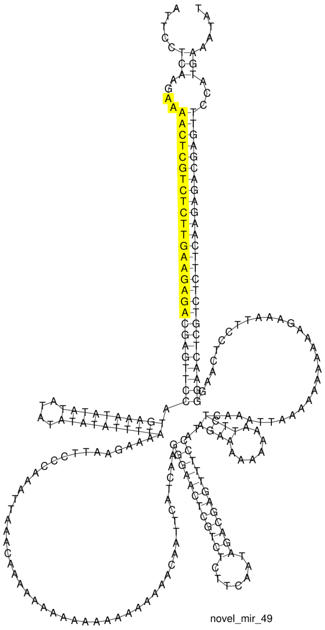

novel_mir_49

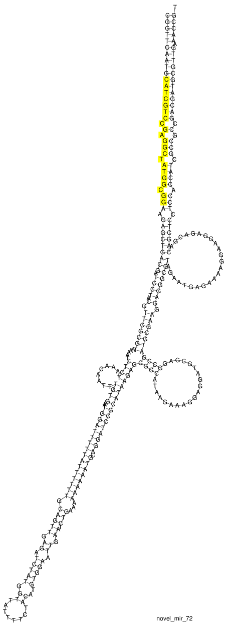

novel_mir_72

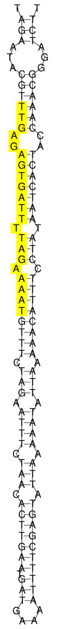

novel_mir_2

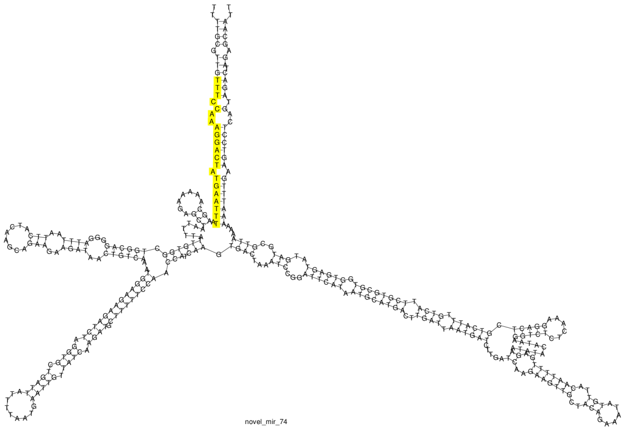

novel_mir_74

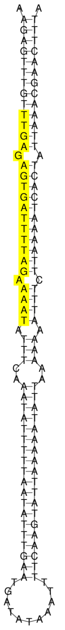

novel_mir_2

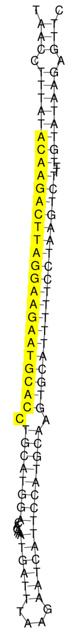

novel_mir_76

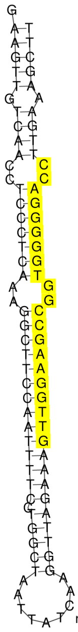

novel_mir_37

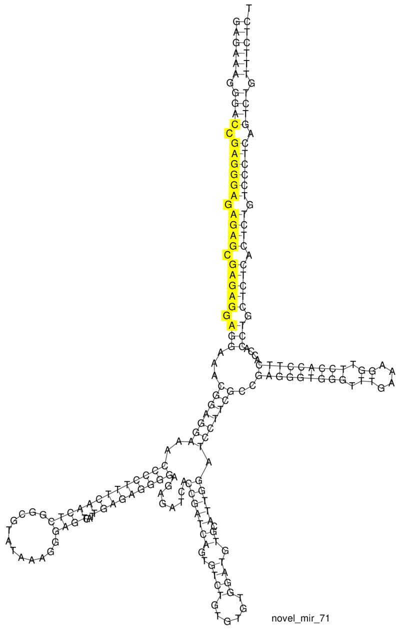

novel_mir_71

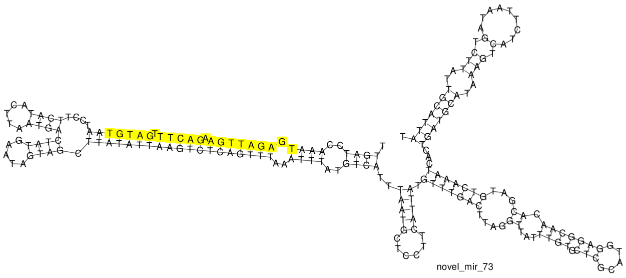

novel_mir_73

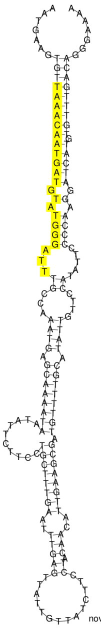

novel_mir_75

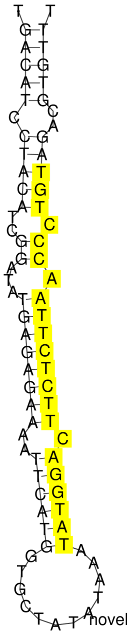

novel_mir_15
