# Supplementary figures and images for: Identification of copper (Cu) stress-responsive grapevine microRNAs and their target genes by high-throughput sequencing
Source: R Soc Open Sci. 2019 Jan 23;6(1):180735. doi: 10.1098/rsos.180735 (PMC6366190; doi:10.1098/rsos.180735)

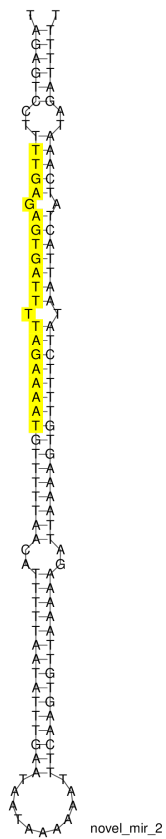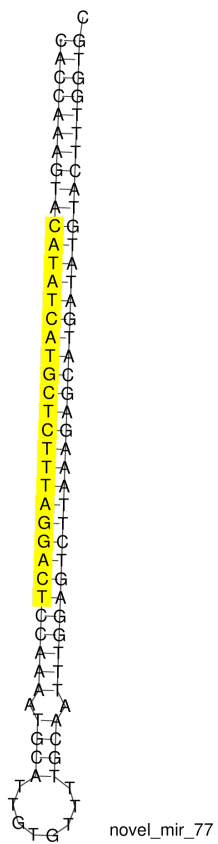

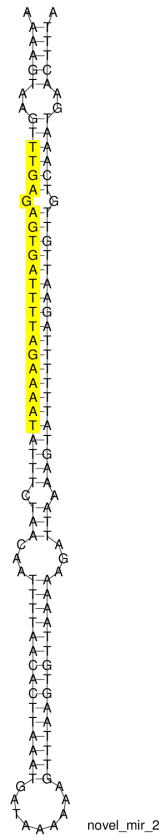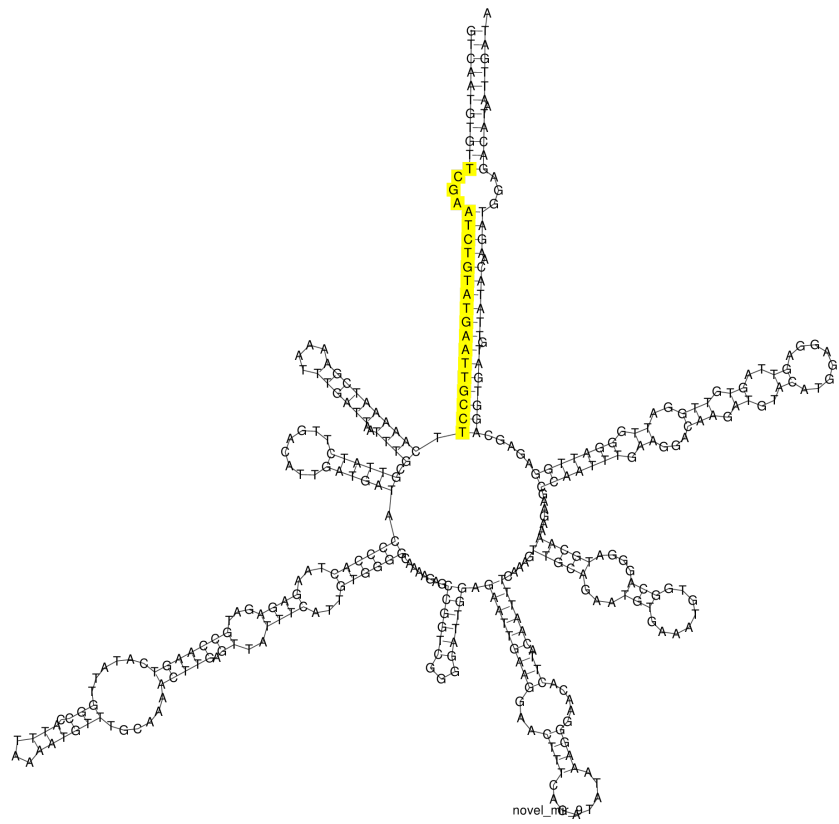

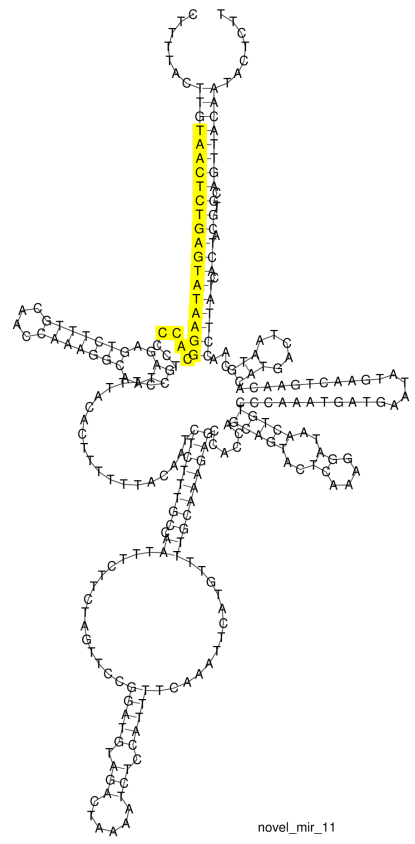

novel\_mir\_11

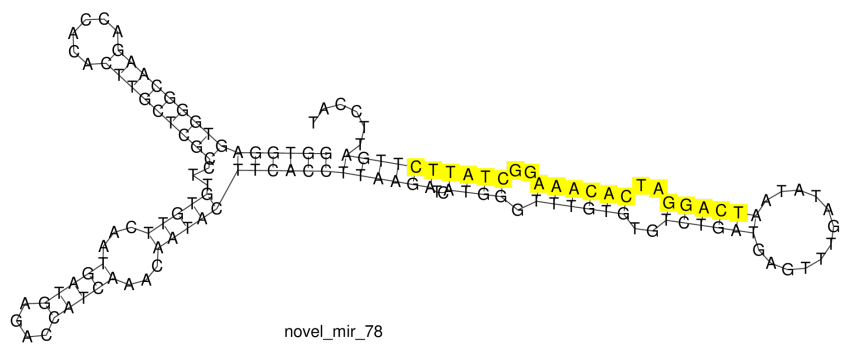

novel\_mir\_78

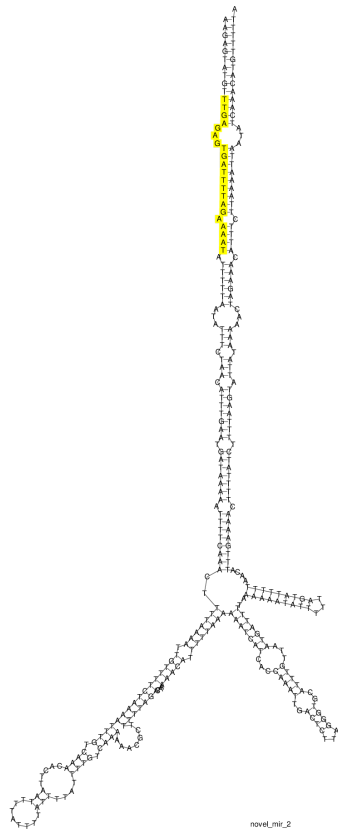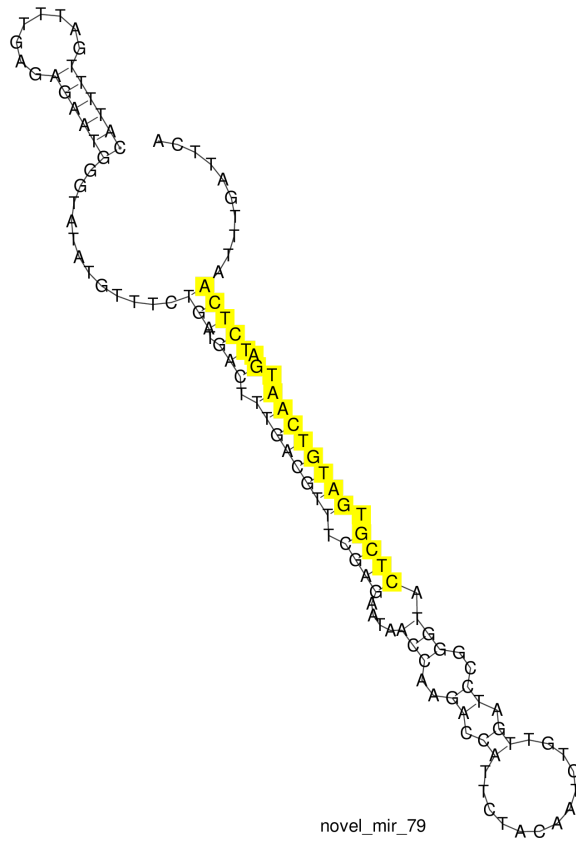

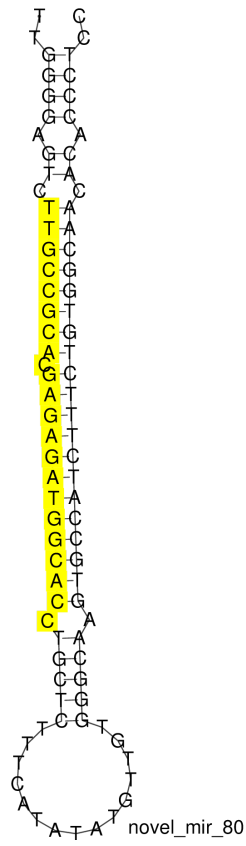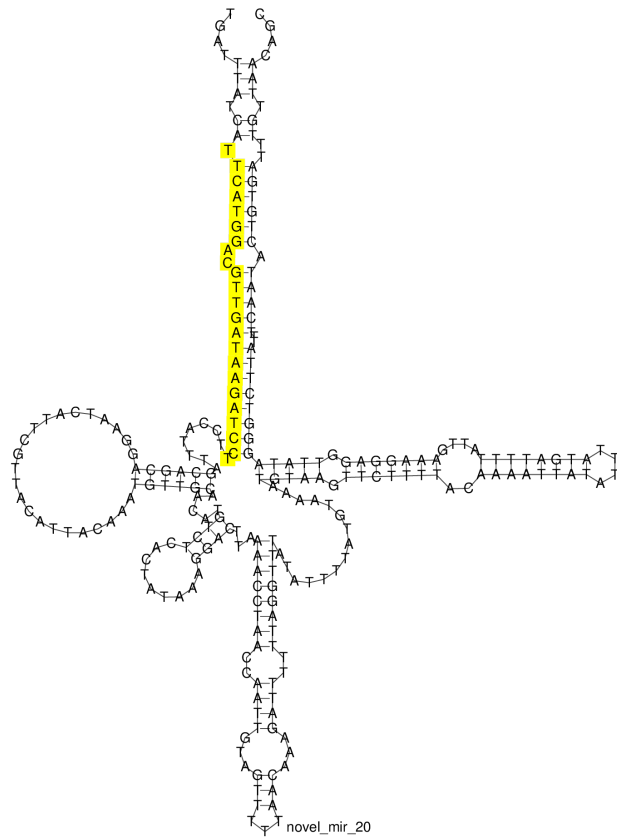

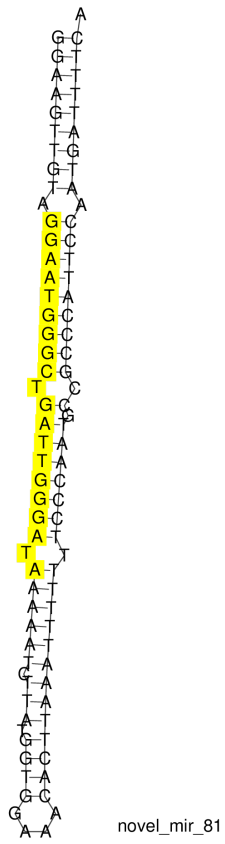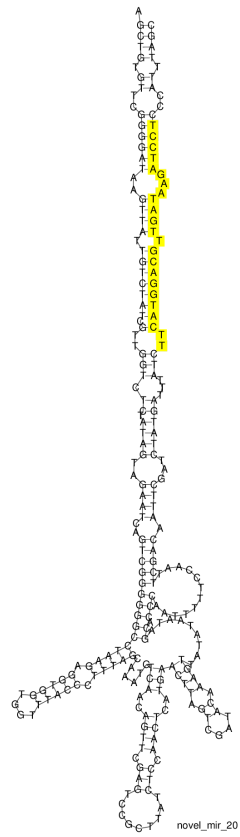

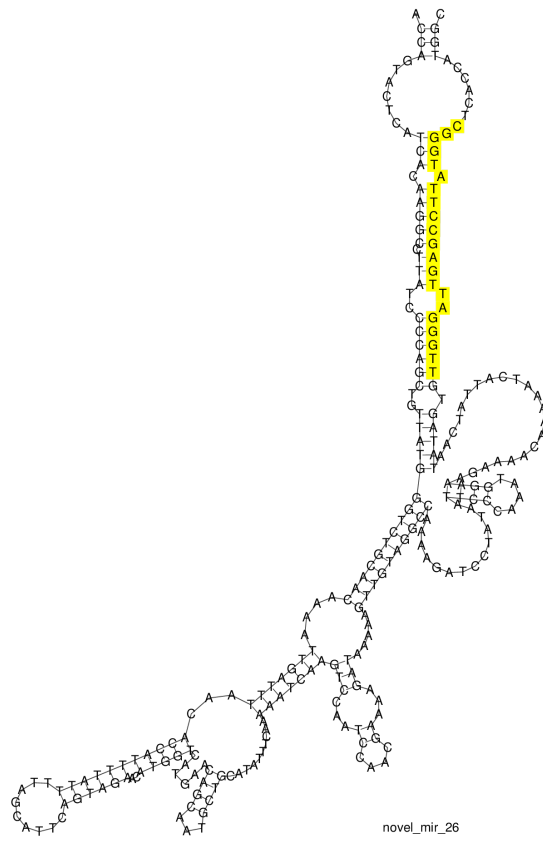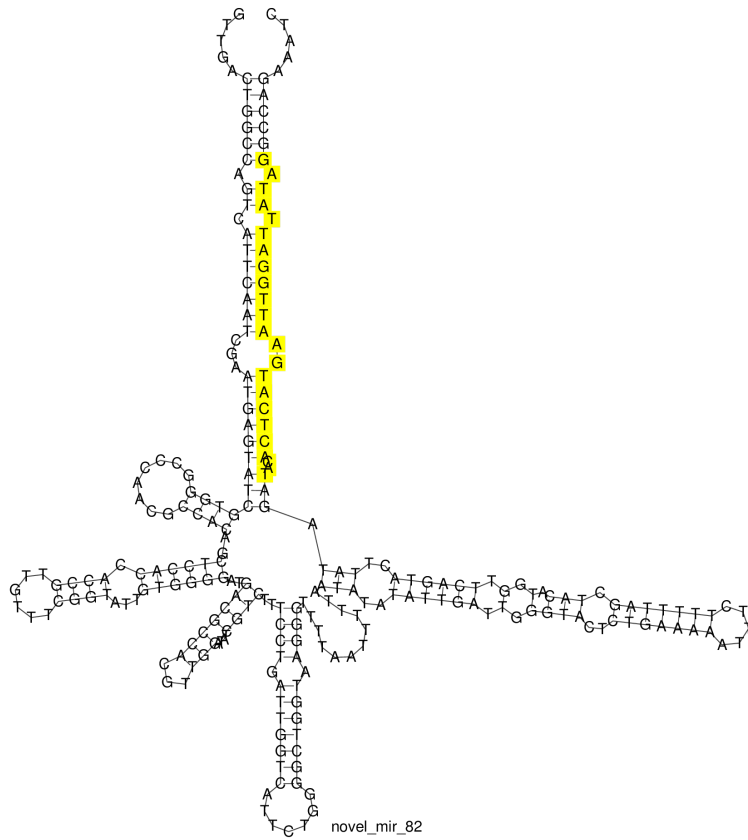

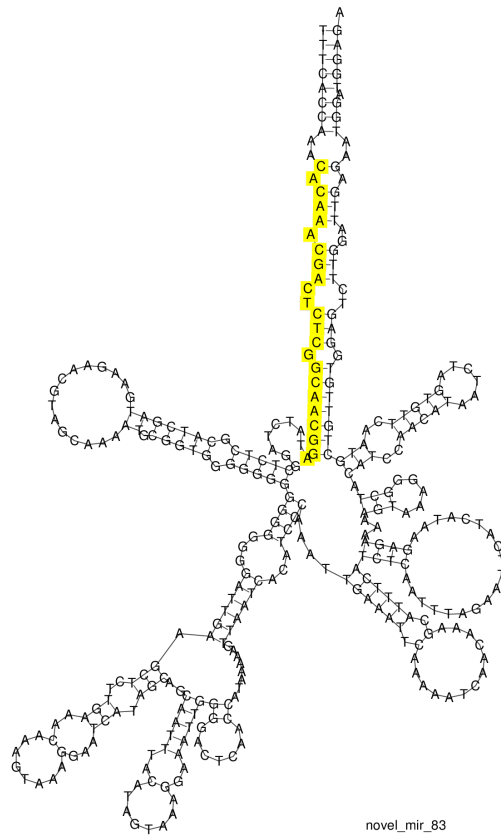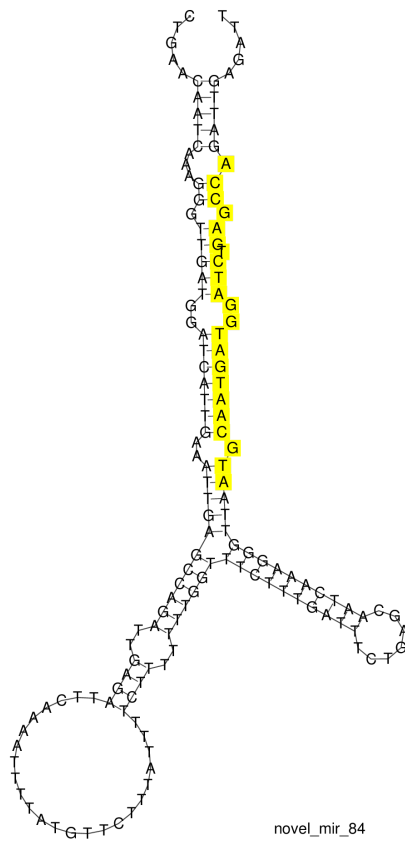

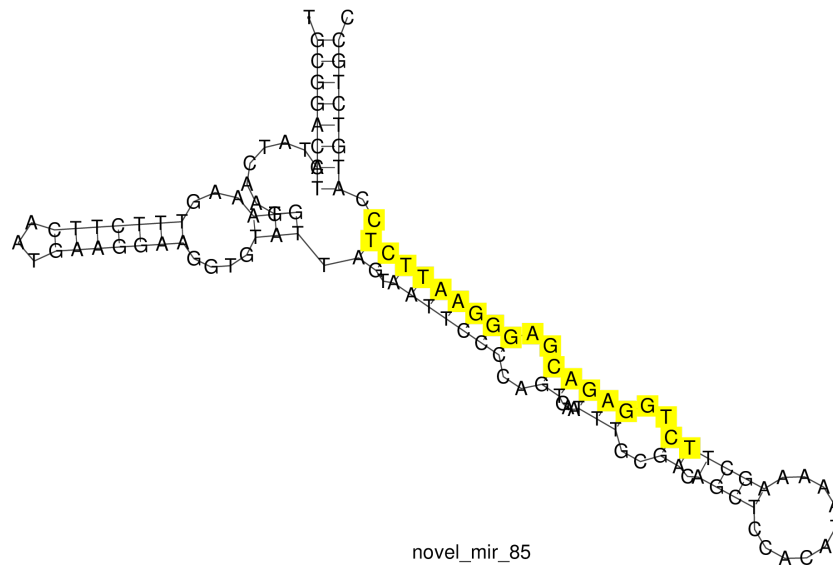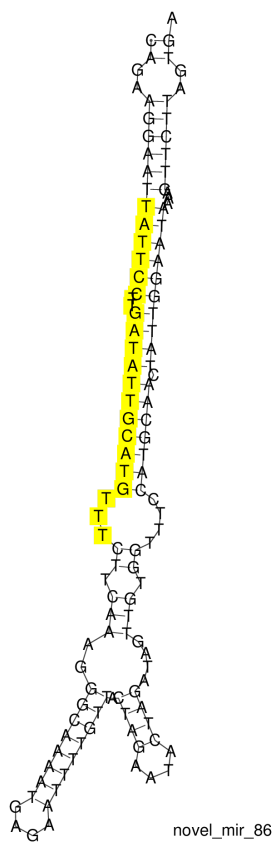

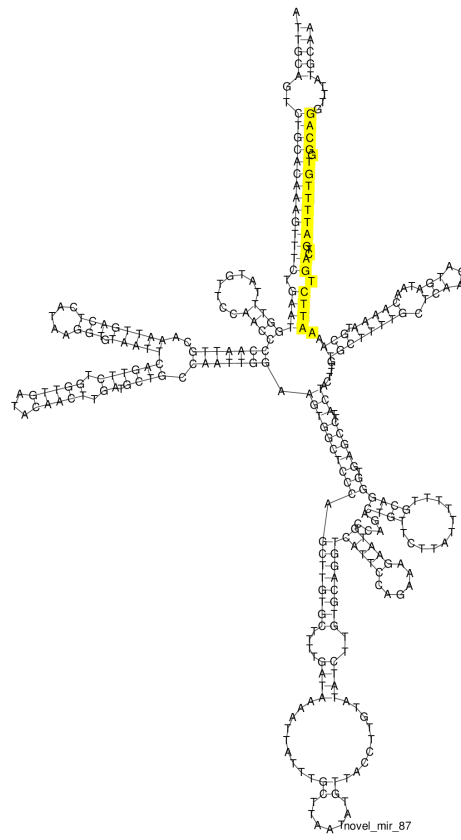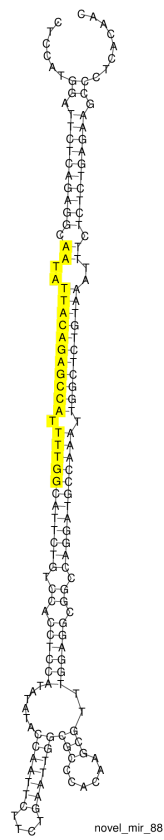



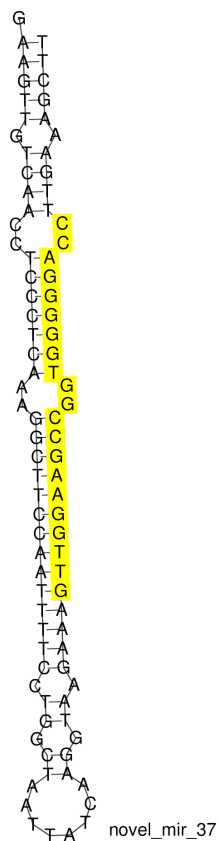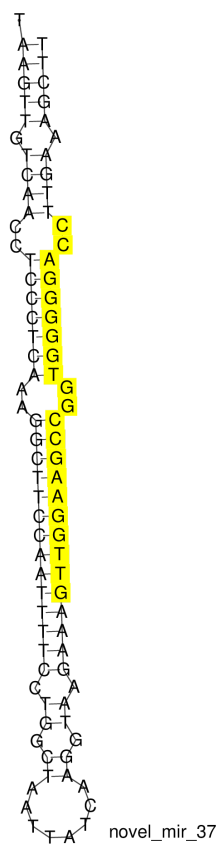

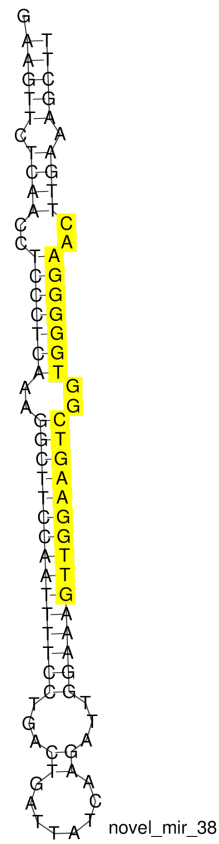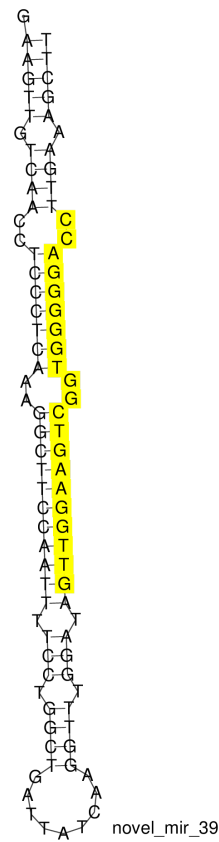

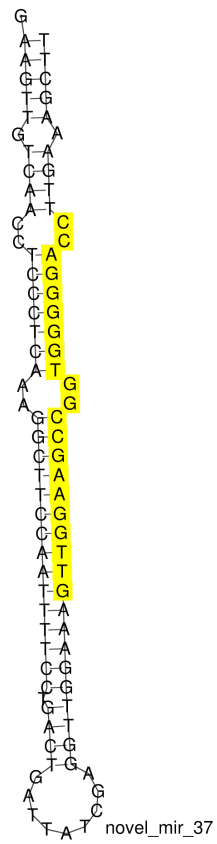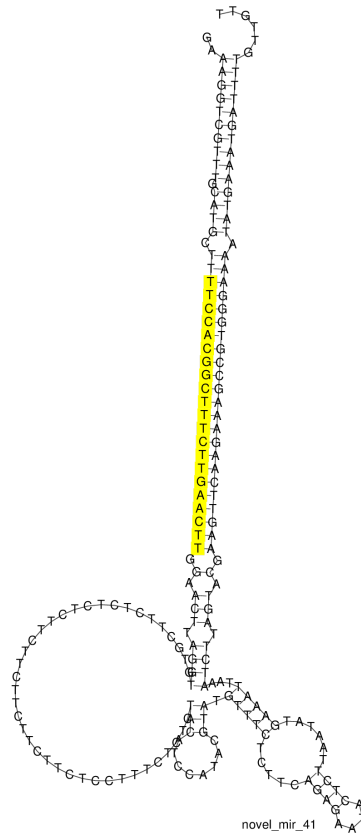

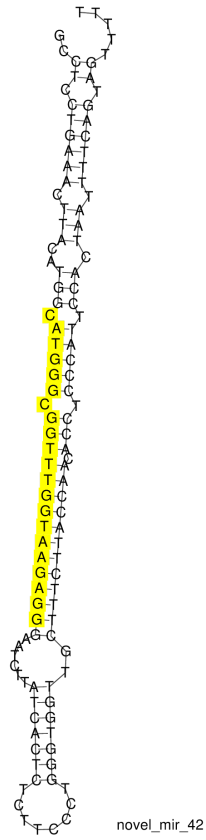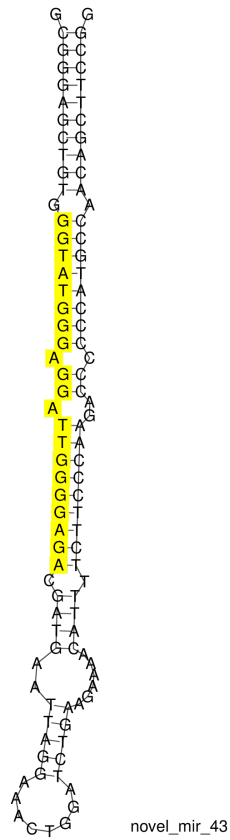



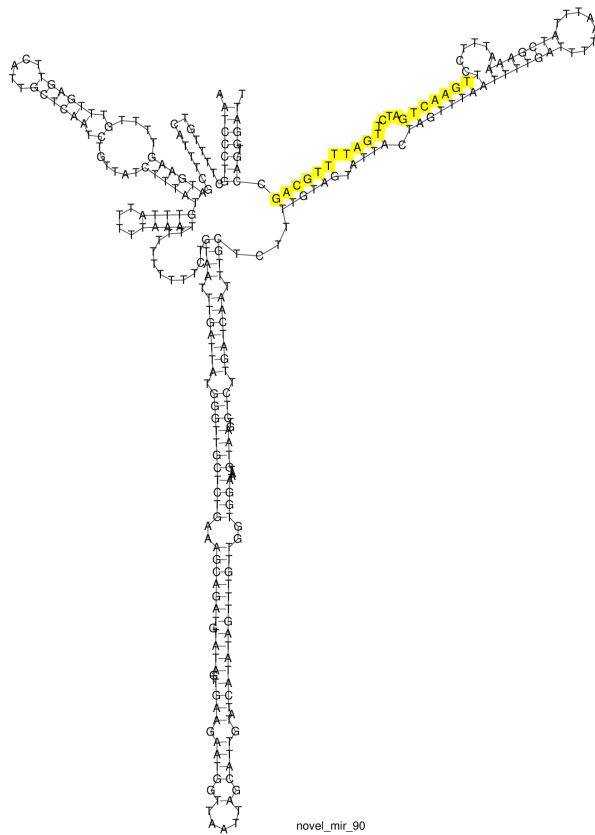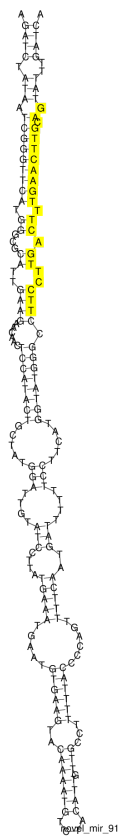

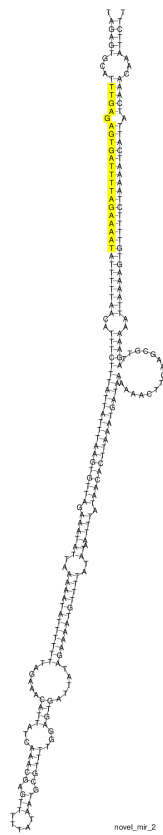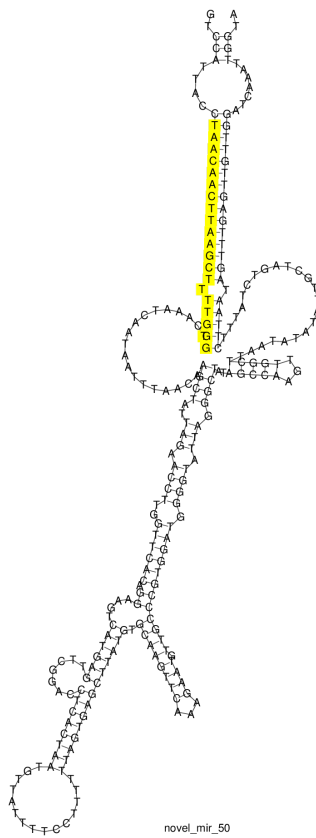

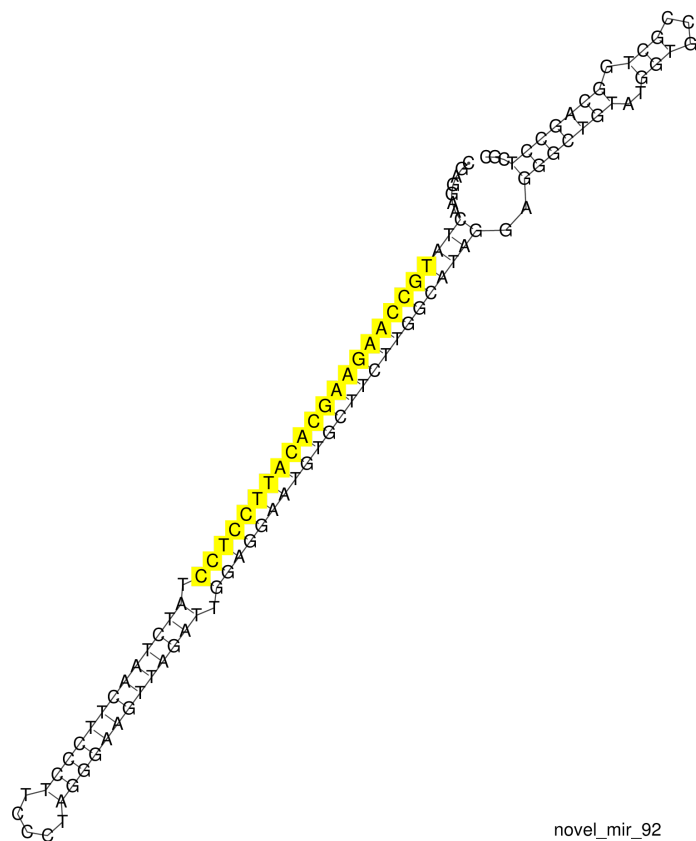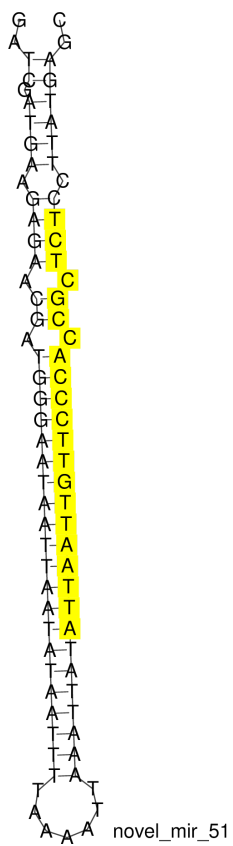

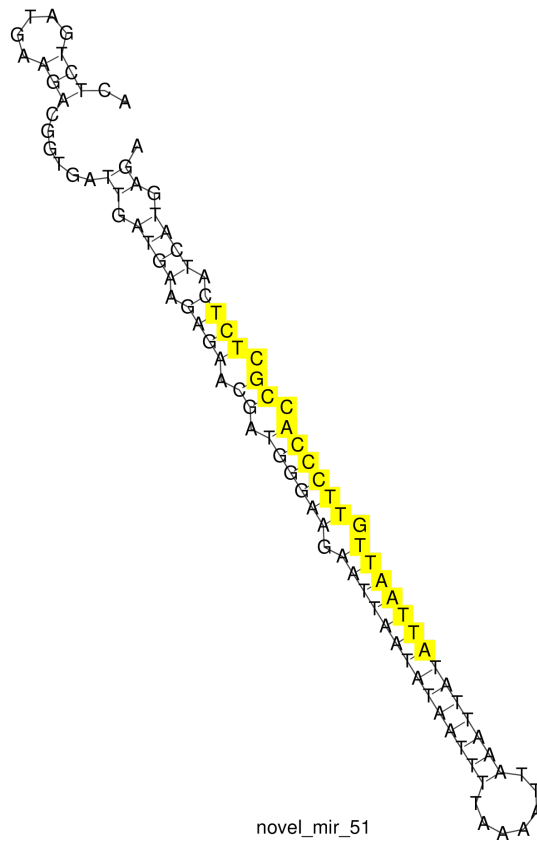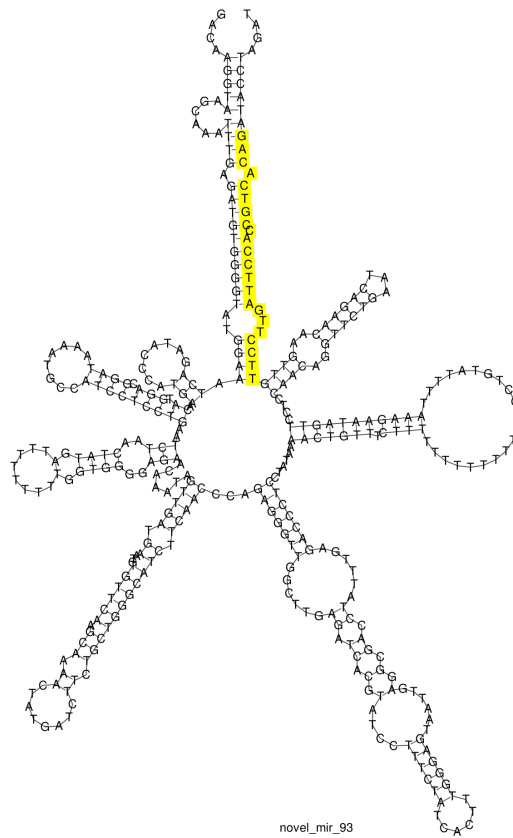



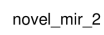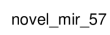

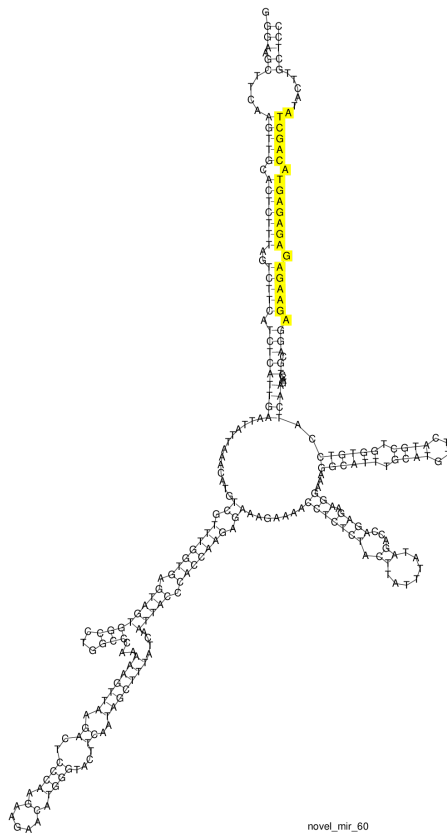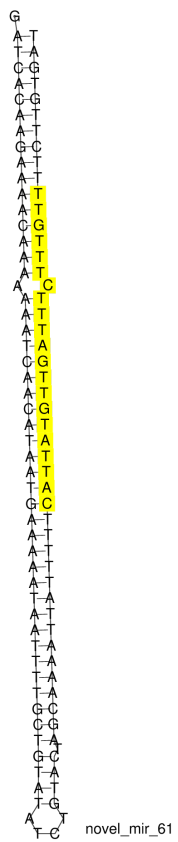

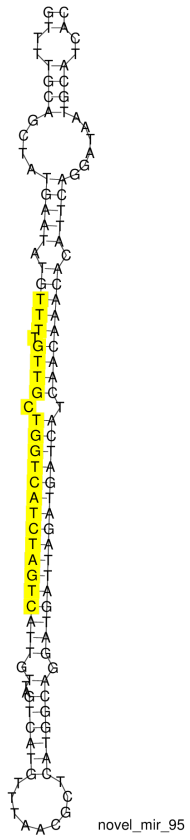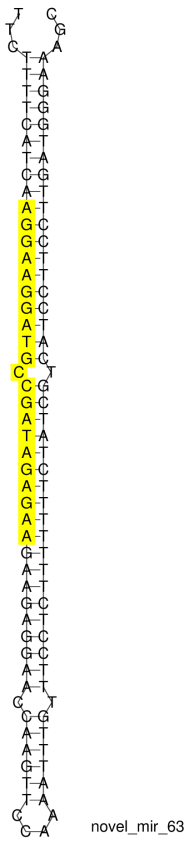

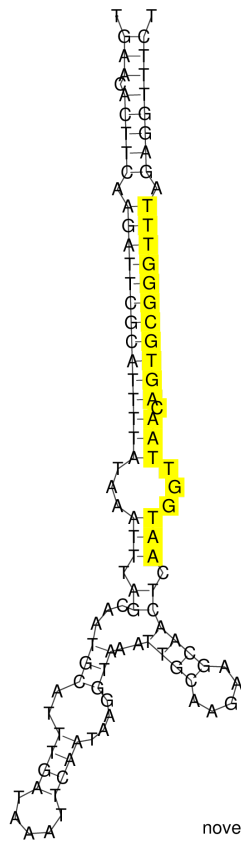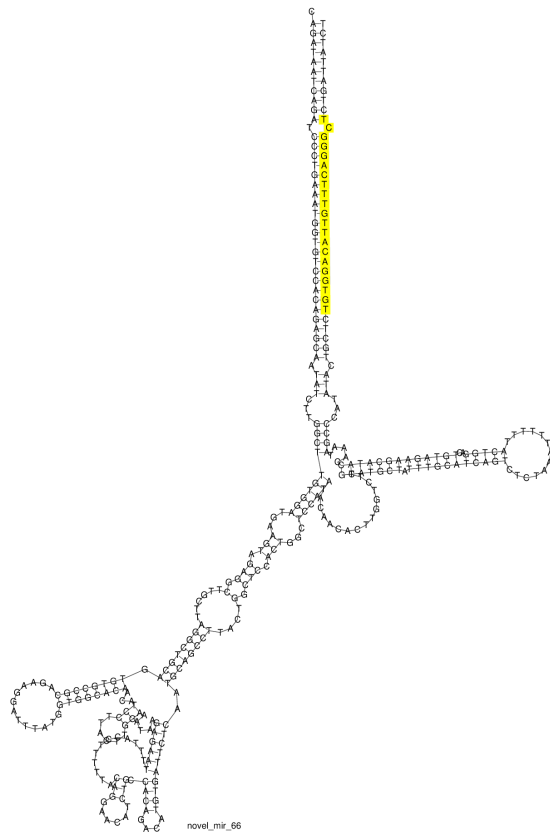

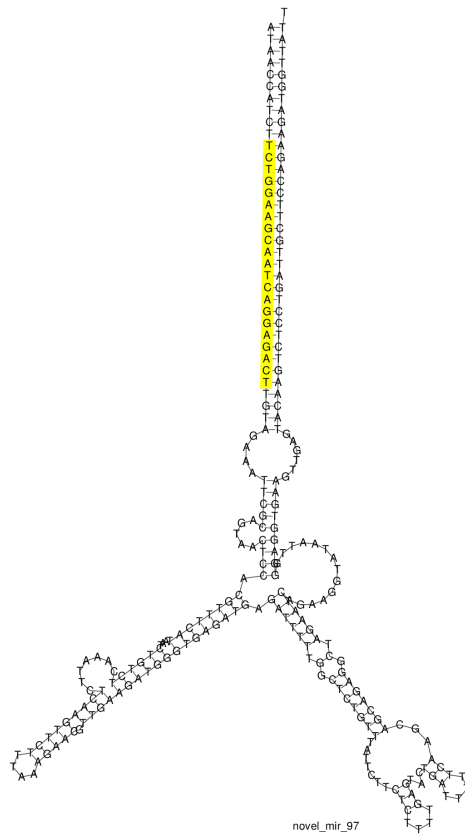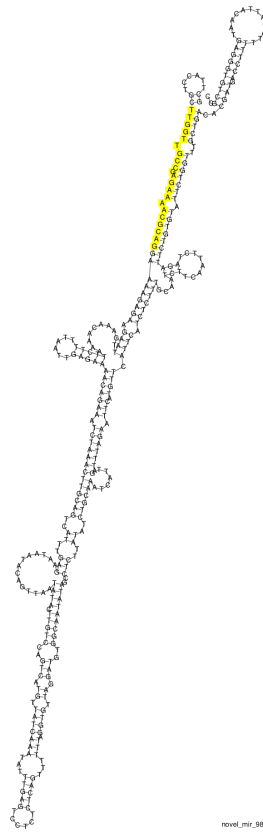

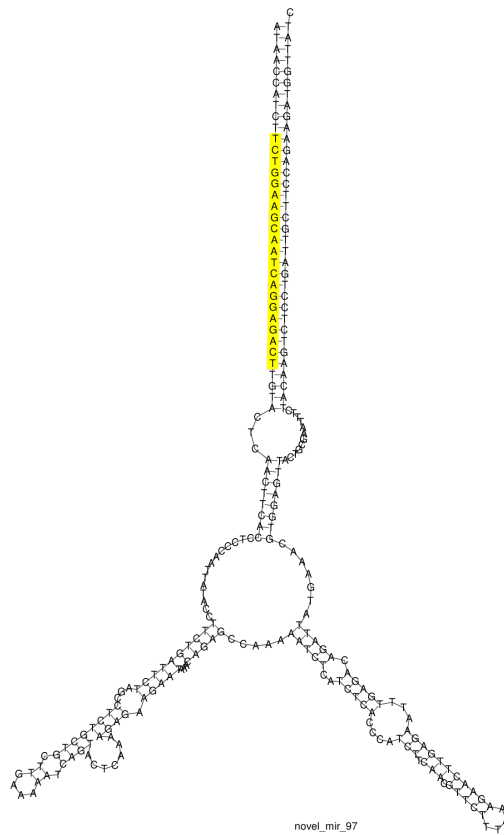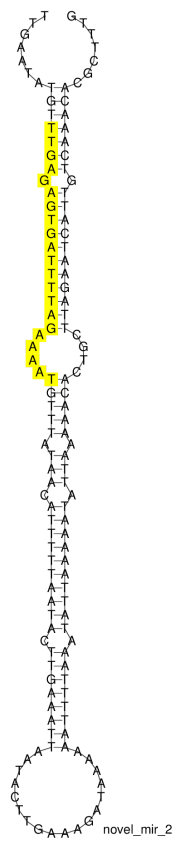

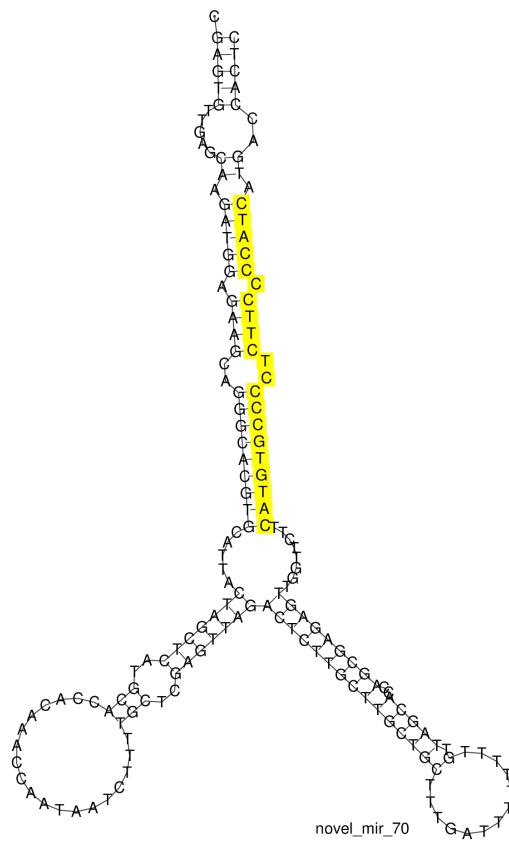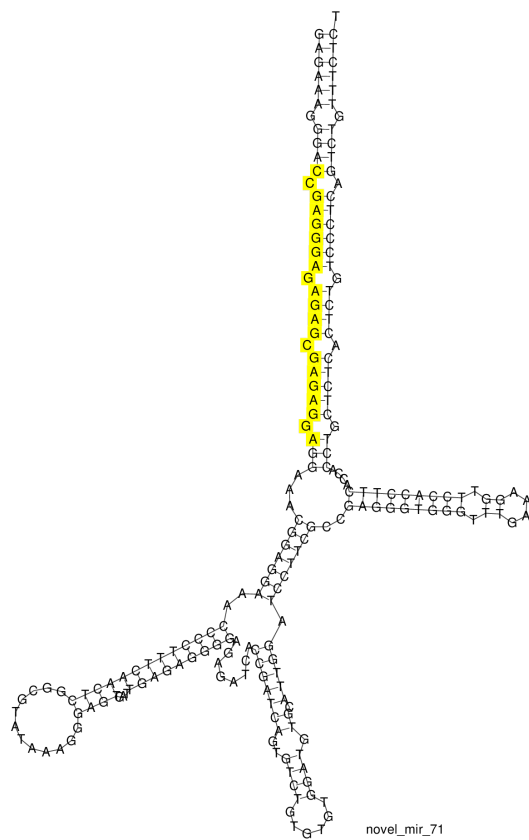

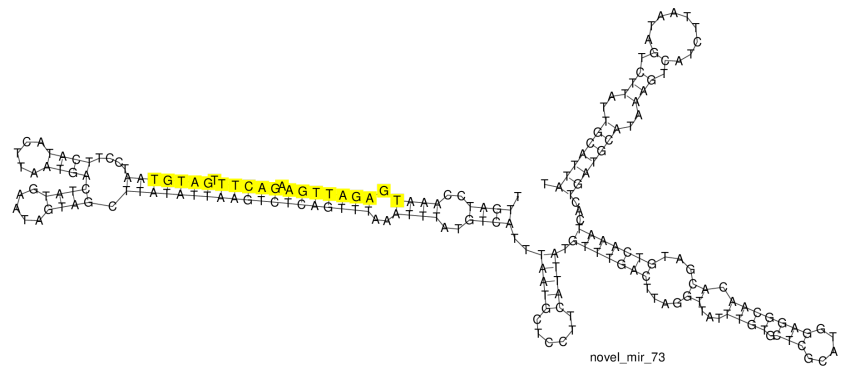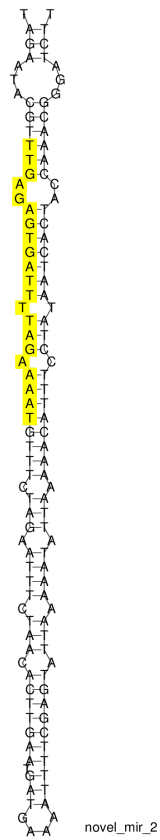

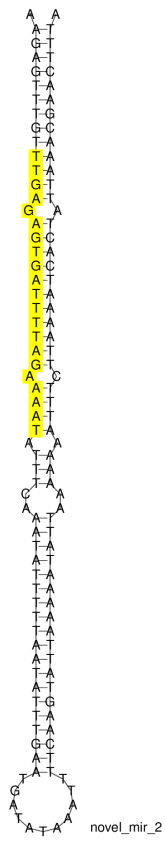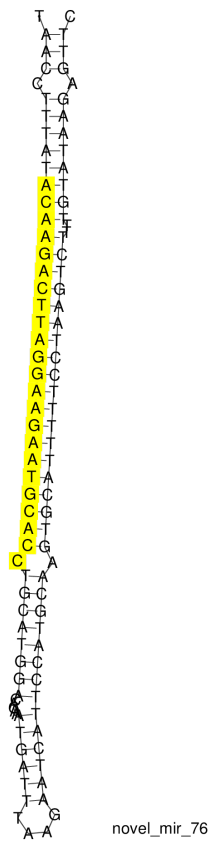

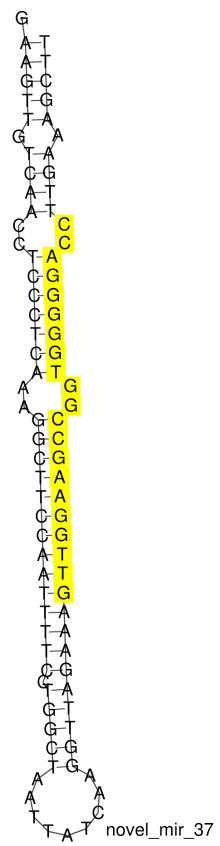

Supplement: Figure S2 [file rsos180735supp2.pdf]

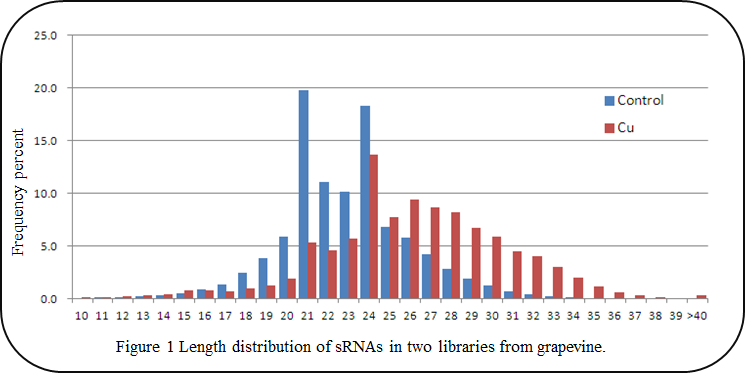

Supplement: Figure S3 [file rsos180735supp3.tif]

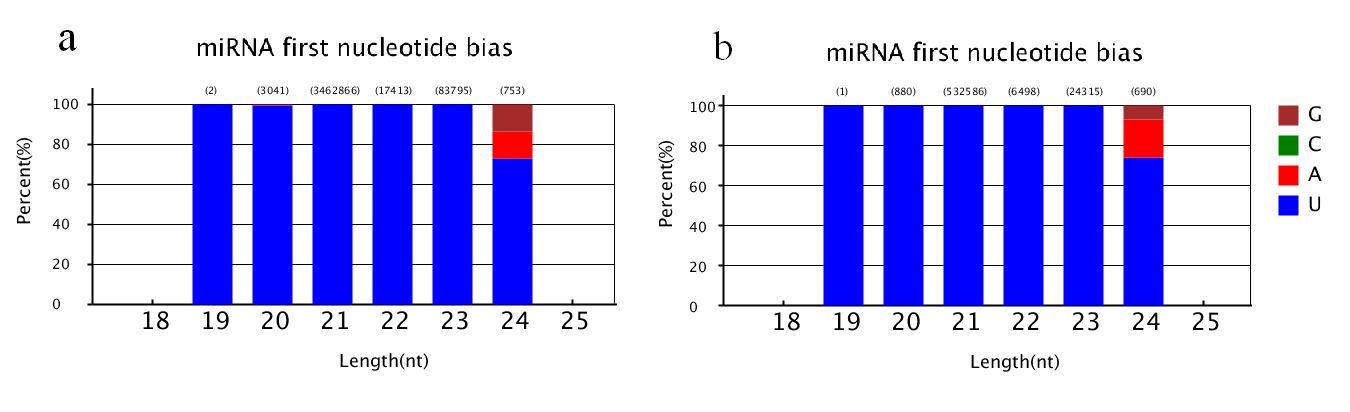

Supplement: Figure S4 [file rsos180735supp4.tif]

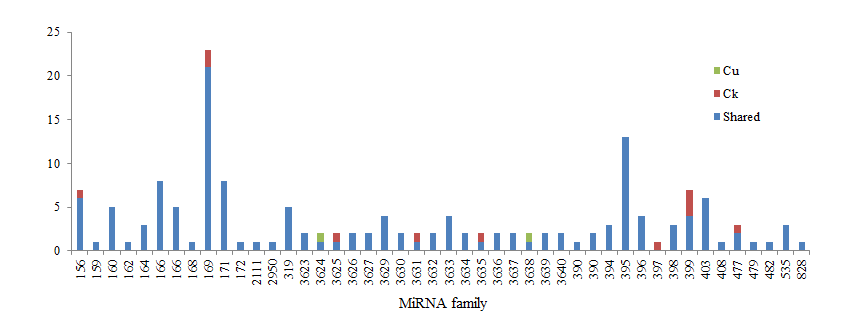

Supplement: Figure S5 [file rsos180735supp5.tif]

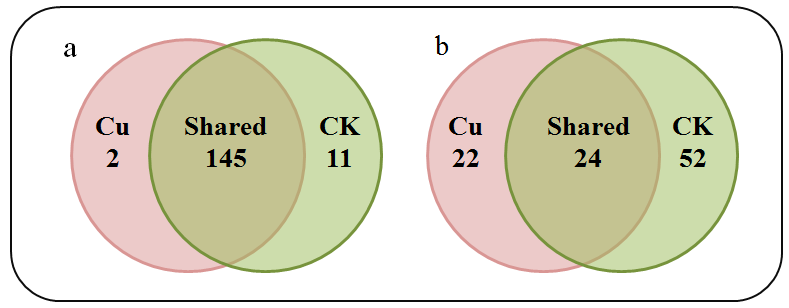

Supplement: Figure S6 [file rsos180735supp6.tif]

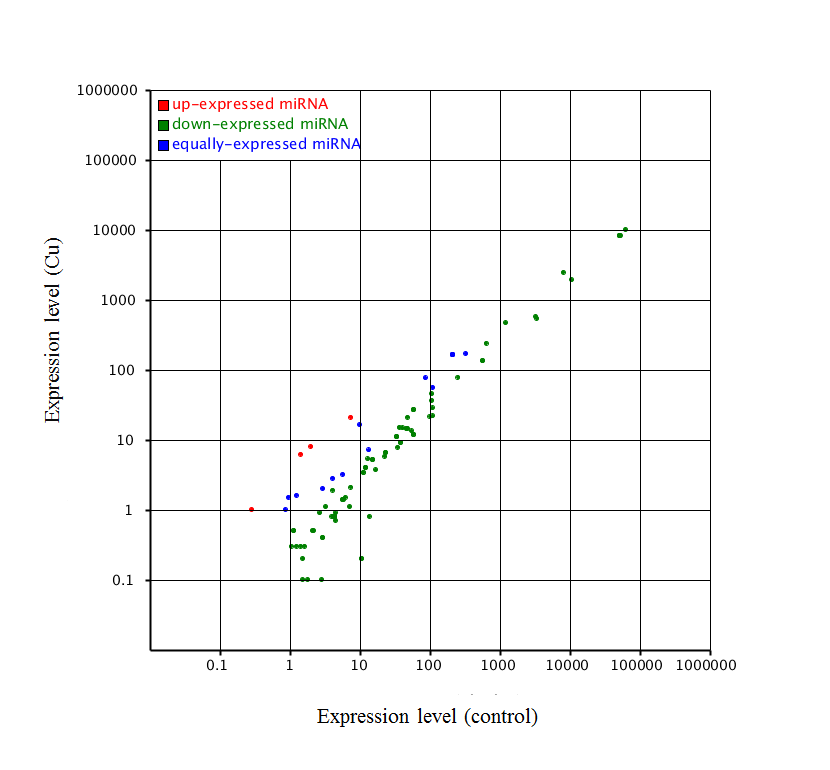

Supplement: Figure S7 [file rsos180735supp7.tif]

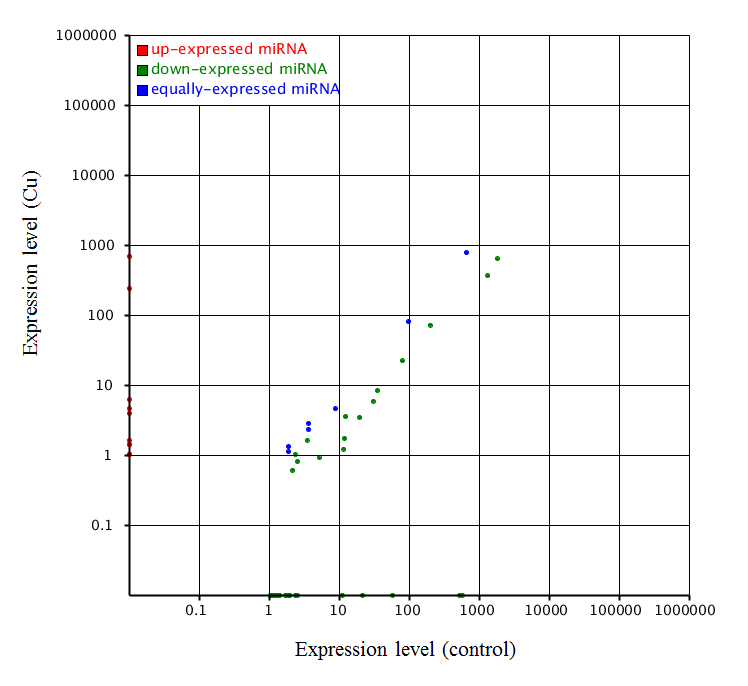

Supplement: Figure S8 [file rsos180735supp8.tif]

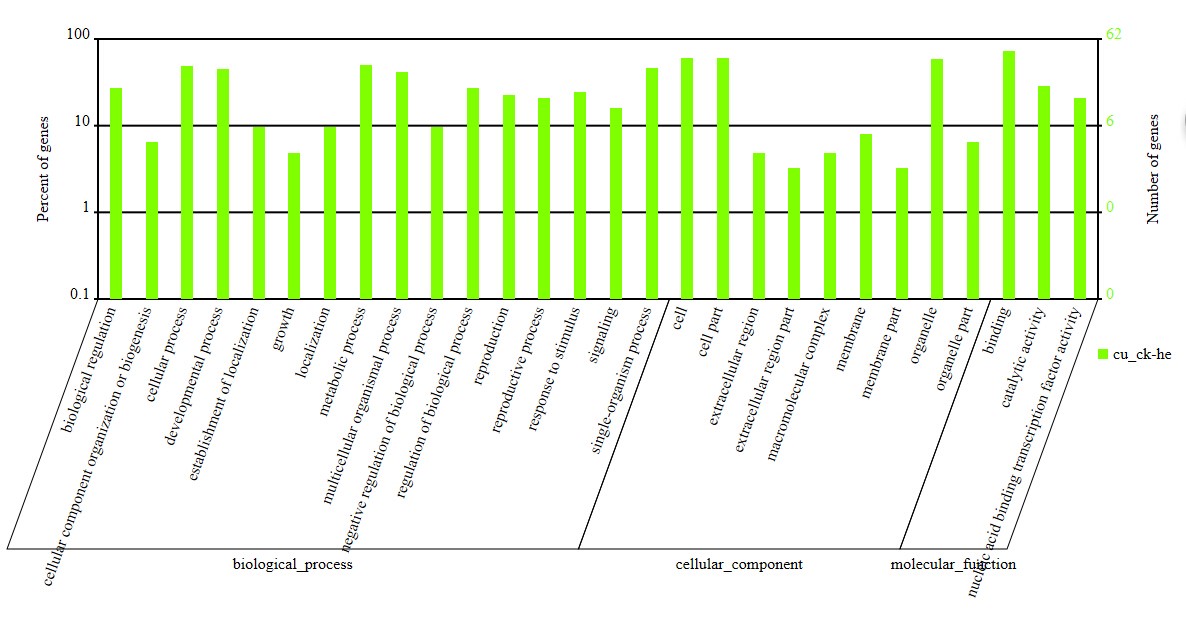

Supplement: Figure S9 [file rsos180735supp9.jpg]

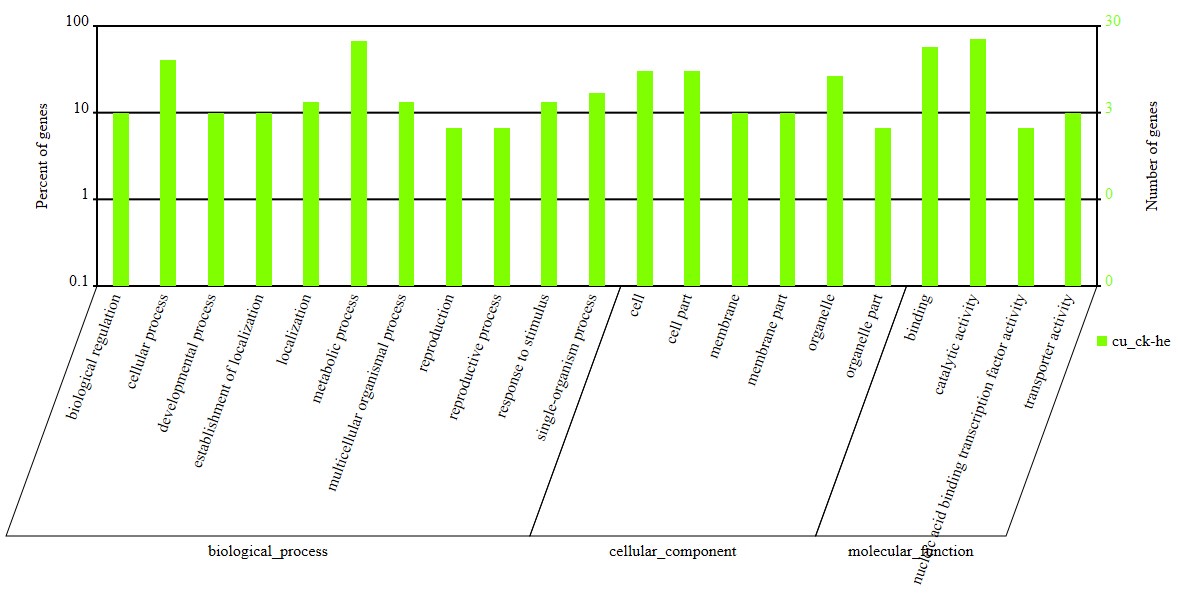

Supplement: Figure S10 [file rsos180735supp10.jpg]
