# Supplementary material for: Identification of copper (Cu) stress-responsive grapevine microRNAs and their target genes by high-throughput sequencing
Source: R Soc Open Sci. 2019 Jan 23;6(1):180735. doi: 10.1098/rsos.180735 (PMC6366190; doi:10.1098/rsos.180735)
Supplement: Table S3 [file rsos180735supp13.docx]

**Table S3 miRNAs and the primers used for qRT-PCR verification.**

| **miRNA** | **Forward primer (5'-3')** | **Reverse primer (5'-3')** |
| --- | --- | --- |
| vvi-miR160a | TGCCTGGCTCCCTGAATGCCA | ATTCTAGAGGCCGAGGCGGCCGACATG |
| vvi-miR172d | AGAATCTTGATGATGCTGCAT | ATTCTAGAGGCCGAGGCGGCCGACATG |
| vvi-miR319b | TTGGACTGAAGGGAGCTCCC | ATTCTAGAGGCCGAGGCGGCCGACATG |
| vvi-miR398b | TGTGTTCTCAGGTCGCCCCTG | ATTCTAGAGGCCGAGGCGGCCGACATG |
| vvi-miR408 | ATGCACTGCCTCTTCCCTGGC | ATTCTAGAGGCCGAGGCGGCCGACATG |
| vvi-miR828a | TCTTGCTCAAATGAGTATTCCA | ATTCTAGAGGCCGAGGCGGCCGACATG |
| novel_mir_37 | GTTGGAAGCCGGTGGGGGACC | ATTCTAGAGGCCGAGGCGGCCGACATG |
| novel_mir_43 | TTCCCAAGACCCCCCATGCCAA | ATTCTAGAGGCCGAGGCGGCCGACATG |
| novel_mir_81 | TCCCAATGCCGCCCATTCCAA | ATTCTAGAGGCCGAGGCGGCCGACATG |
| 5.8 S | CTCGGCAACGGATATCTCGGCTCT | CTAATGGCTTGGGGCGCAACTTG |
