# Supplementary material for: Identification of copper (Cu) stress-responsive grapevine microRNAs and their target genes by high-throughput sequencing
Source: R Soc Open Sci. 2019 Jan 23;6(1):180735. doi: 10.1098/rsos.180735 (PMC6366190; doi:10.1098/rsos.180735)
Supplement: Table S4 [file rsos180735supp14.docx]

**Table S4 Target genes and the primers used for qRT-PCR verification.**

| **Target genes** | **Forward primer (5'-3')** | **Reverse primer (5'-3')** |
| --- | --- | --- |
| *GSVIVT01008950001* | GTCTTCTCCAAACCTTCTGT | TTCCTTGTCATCATCACCAT |
| *GSVIVT01022081001* | CTACTTCCTGCGGTGGTGCT | TTACTGCTTCGCTTCCTCTG |
| *GSVIVT01025548001* | ACGAGAAGCAGTAACCAACT | AGTGTTGCTGAAGTAGAGTT |
| *GSVIVT01012447001* | ATTCCTCTACTGTTACTCCT | GGTGGCGGTGAAGAAGATGT |
| *GSVIVT01010003001* | TCACTCTCACAACTTCAGCT | CAGTTAGCGGAGTTGAAGGT |
| *GSVIVT01016765001* | TGGCTGAGGTTATTGAAGTC | CCACTTTGCTTCTCCTGCTT |
| *GSVIVT01030338001* | CTCTGCTCCTGTTCCAGAAT | GTGAGAGGAAGGAATCGGAC |
| *GSVIVT01032824001* | TAGAAGAGGTGATGGCAATT | ATCCTCCATTGTTTCTTCTC |
| *GSVIVT01022941001* | CGGTTGGTCTAAATGGAAGT | CACATTTCGCTATTCAACAC |
| *Actin* | GGAATGGGACAGAAGGAT | CAGTCAGGAGAACAGGGT |
