# Supplementary material for: Identification of copper (Cu) stress-responsive grapevine microRNAs and their target genes by high-throughput sequencing
Source: R Soc Open Sci. 2019 Jan 23;6(1):180735. doi: 10.1098/rsos.180735 (PMC6366190; doi:10.1098/rsos.180735)
Supplement: Table S5 [file rsos180735supp15.docx]

| **Table S5 Primer sequences for 5’-RLM-RACE of the 3’ products of miRNA cleaved target genes.** | | | |
| --- | --- | --- | --- |
| **miRNA ID** | **Target gene** | **Forward primer sequences (5'-3')**  **(common primer)** | **Reverse primer sequences (5'-3')**  **(specific primers)** |
| vvi-miR319f | *GSVIVT01012447001* | AGGACACTGACATGGACTGAAGGAGTAG | CCATAGTCTTCCCATTCTGT |
| vvi-miR535a | *GSVIVT01006378001* | AGGACACTGACATGGACTGAAGGAGTAG | CGAACCCATTTATTTCAGCT |
| novel_mir_11 | *GSVIVT01022931001* | AGGACACTGACATGGACTGAAGGAGTAG | TCACCCACCTCTTCCATTGT |
| novel_mir_97 | *GSVIVT01022165001* | AGGACACTGACATGGACTGAAGGAGTAG | CGAGCAACCGACACACAAAT |
